# Olmesartan-Loaded PLGA Nanoparticles Attenuate Methotrexate-Induced Kidney Injury: Association with the AT1R/ERK1/2 Signaling Axis

**DOI:** 10.3390/ph19091462

**Published:** 2026-09-15

**Authors:** Omar M. Alawad, Norhan Tantawy, Soha Elsalhy, Asmaa A. Ahmed, Shahira Nofal, Eman M. Raafat

**Affiliations:** 1 Inspection Department, Ministry of Health Branch in Madinah, Madinah 41311, Saudi Arabia; oma2007sa@gmail.com; 2 Compliance Department, Ministry of Health Branch in Madinah, Madinah 41311, Saudi Arabia; 3 Department of Pharmaceutics and Industrial Pharmacy, Faculty of Pharmacy, Capital University (Formerly Helwan University), Cairo 11795, Egypt; norhan.eltantawy@pharm.capu.edu.eg; 4 Department of Pharmaceutics, College of Pharmaceutical Sciences and Drug Manufacturing, Misr University for Science and Technology (MUST), Giza 12585, Egypt; soha.mohamed@must.edu.eg; 5 Department of Pharmacology and Toxicology, Faculty of Pharmacy, Capital University (Formerly Helwan University), Cairo 11795, Egypt; shahira.nofal@pharm.capu.edu.eg (S.N.); eman.raffat@pharm.capu.edu.eg (E.M.R.)

**Keywords:** methotrexate, olmesartan, PLGA, angiotensin II type 1 receptor, ERK1/2

## Abstract

**Background/Objectives:** Methotrexate (MTX) is an effective antineoplastic and immunosuppressive agent whose clinical use can be limited by nephrotoxicity. Increasing evidence suggests that dysregulated angiotensin II-mediated signaling contributes to MTX-induced renal injury. Olmesartan (OLM), an angiotensin II type 1 receptor (AT1R) blocker, possesses renoprotective properties; however, its therapeutic efficacy may be limited by suboptimal pharmacokinetics and tissue deli\very. This study aimed to develop OLM-loaded poly(lactic-co-glycolic acid) (PLGA) nanoparticles (OLM-PLGA) and investigate their nephroprotective efficacy and underlying molecular mechanisms in MTX-induced nephrotoxicity. **Methods:** OLM-PLGA nanoparticles were prepared and evaluated for their physicochemical characteristics. The in vivo nephroprotective effects of OLM-PLGA were investigated in male *Sprague–Dawley* rats, which were allocated into four groups: OLM (10 mg/kg), OLM-PLGA (10 mg/kg), MTX-treated, and normal control. Renal function, oxidative stress, inflammation, apoptosis, and fibrosis were assessed, together with renal gene expression of AT1R and extracellular signal-regulated kinase 1/2 (ERK1/2). **Results**: Compared with free OLM, OLM-PLGA provided superior protection against MTX-induced renal dysfunction and oxidative stress, as evidenced by improved renal function and enhanced antioxidant defense. OLM-PLGA also exerted greater anti-inflammatory and anti-apoptotic effects and attenuated renal fibrotic changes, accompanied by reduced renal expression of α-smooth muscle actin and collagen. These protective effects were associated with decreased AT1R gene expression and suppression of downstream ERK1/2 signaling. **Conclusions**: PLGA-based nanoencapsulation enhanced the nephroprotective efficacy of OLM against MTX-induced nephrotoxicity and was associated with the modulation of the AT1R/ERK1/2 signaling axis. OLM-PLGA may therefore represent a promising nanotherapeutic approach for improving OLM delivery and renal protection during MTX treatment.

## 1. Introduction

Owing to its ability to inhibit folate-dependent pathways, methotrexate (MTX) serves as a well-established therapeutic agent in both cancer management and immunosuppressive therapy for rheumatoid arthritis, psoriasis, and several autoimmune disorders. Its pharmacological activity mainly depends on the inhibition of dihydrofolate reductase, leading to suppression of DNA synthesis and cellular proliferation [1,2]. Despite its therapeutic efficacy, the clinical use of MTX in treating all types of cancer is often challenged by the risk of serious adverse effects, particularly nephrotoxicity, because the kidneys are the major route for MTX excretion. High-dose MTX therapy may induce acute kidney injury through precipitation of MTX and its metabolites within renal tubules in addition to direct tubular toxicity [3].

Methotrexate (MTX)-induced nephrotoxicity is primarily mediated through oxidative stress, inflammation, and apoptosis [3]. MTX increases reactive oxygen species (ROS) generation, disrupting redox homeostasis and causing lipid peroxidation, mitochondrial dysfunction, and cellular damage [4]. Excessive ROS also activate the nuclear factor-kappa B///Tumor Necrosis Factor-Alpha (NF-κB/TNF-α) inflammatory signaling pathway, promoting inflammatory cell infiltration and aggravating tubular injury [4,5]. Additionally, MTX triggers apoptosis by disturbing mitochondrial integrity, leading to a shift in apoptotic regulatory proteins, with increased Bax and decreased Bcl-2 levels, leading to excessive renal tubular epithelial cell death and the subsequent deterioration of renal structure and function [6].

Renal fibrosis represents a principal pathological consequence of continuous renal injury and chronic inflammatory responses. Chronic activation of inflammatory and oxidative pathways stimulates fibroblast activation and extracellular matrix accumulation within renal tissues [7]. Alpha-smooth muscle actin (α-SMA) is widely recognized as an indicator of myofibroblast activation, while increased collagen deposition reflects the progression of renal fibrosis. These fibrotic alterations ultimately contribute to structural remodeling and the chronic deterioration of kidney function [8].

Extracellular signal-regulated kinase 1/2 (ERK1/2) are critically contributing to the pathogenesis of renal injury and fibrosis [9]. ERK1/2 activation occurs in response to oxidative stress and inflammatory mediators and participates in the regulation of cell survival, apoptosis, and fibrogenesis. Dysregulation of ERK1/2 signaling has been associated with enhanced inflammatory responses and progression of diseases [10,11].

Emerging evidence suggests that activation of angiotensin II type 1 receptor (AT1R) signaling may contribute to renal injury and nephrotoxicity [12,13,14]. Angiotensin II is involved in promoting both oxidative stress and inflammatory processes through stimulation of NADPH oxidase [15] and NF-κB activation [16], thereby aggravating renal injury and dysfunction. Moreover, Angiotensin II signaling enhanced profibrotic signaling pathways via AT1 receptor-dependent phosphorylation of ERK1/2 [17,18]. Therefore, blockade of AT1 receptors may provide nephroprotective effects through the attenuation of oxidative stress, inflammation, apoptosis, and fibrosis.

Olmesartan (OLM) is a selective angiotensin II receptor blocker that exerts potent antihypertensive [19] and nephroprotective activity [20,21]. Beyond its blood pressure-lowering action, OLM possesses antioxidant [21,22], anti-inflammatory [22,23], anti-apoptotic [24,25], and antifibrotic properties [26,27]. The therapeutic efficacy of orally administered OLM is restricted by its poor aqueous solubility, inadequate bioavailability (~26.0%), extensive first-pass metabolism, and unwanted systemic effects [28]. These limitations have encouraged the development of PLGA-based nanoformulation approaches as potential strategies to improve drug solubility, controlled release, delivery efficiency, and therapeutic performance. Recent studies have highlighted the potential of nanoparticle-based systems to improve drug delivery while emphasizing the importance of formulation characteristics, controlled drug release, and safety considerations [29,30].

**Figure 1:**
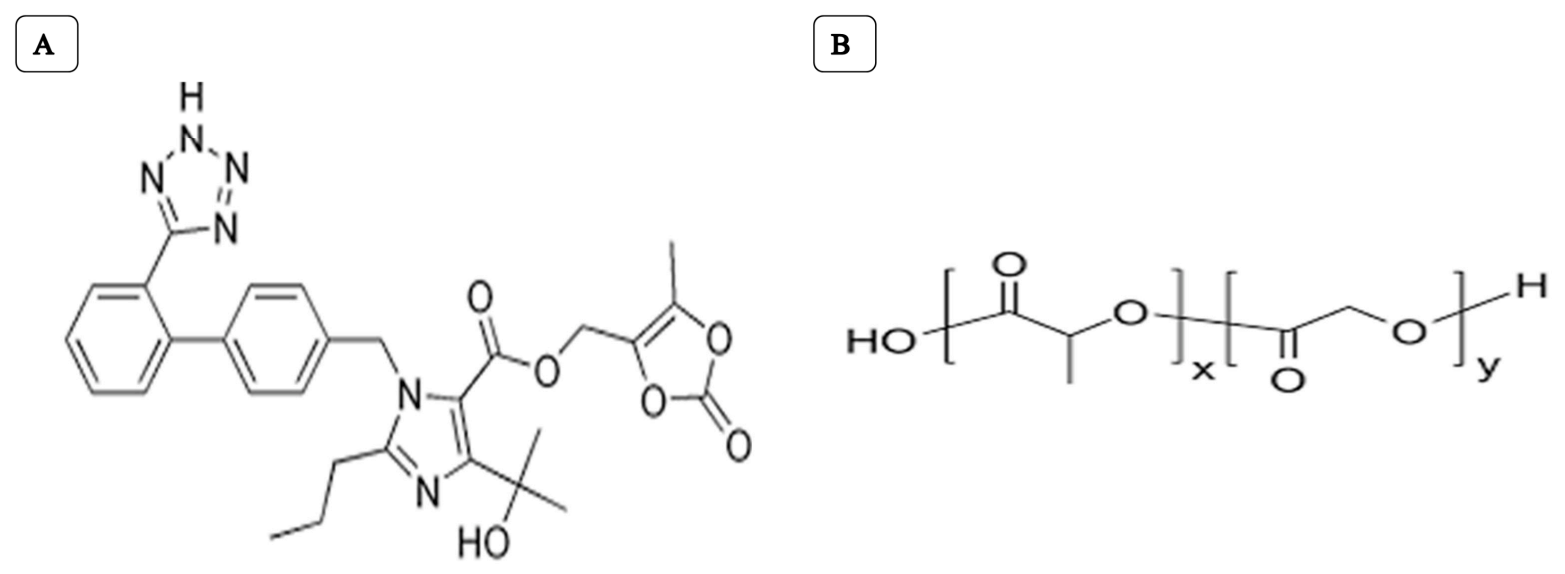
Structure of (**A**) olmesartan (OLM) and (**B**) poly lactic-*co*-glycolic acid (PLGA), where x is the number of lactic acid units and y is number of glycolic acid units.

Employing nanoparticles for drug delivery provides a feasible approach to bypassing these challenges. Biodegradable poly (lactide-co-glycolic) acid (PLGA) nanoparticles (PLGA NPs) have lately emerged as an effective drug delivery system. The molecular structure of OLM, along with the polymeric structure of PLGA used for nanoparticle preparation, is presented in Figure 1. PLGA nanoparticles have significant biocompatibility and have received FDA approval for diagnostic purposes, parenteral treatment, and several therapeutic applications, including oncology, cardiovascular disorders, neurological illnesses, vaccinations, and tissue engineering [31]. PLGA nanoparticles may improve the physicochemical properties of poorly soluble drugs and provide controlled drug release, while their formulation characteristics can influence drug stability, cellular uptake, and tissue distribution. However, the extent to which these advantages translate into prolonged systemic exposure or enhanced accumulation in specific tissues depends on nanoparticle composition, particle size, surface characteristics, administration route, and other formulation-related factors [32,33]. Moreover, a previous report documented that PLGA nanoparticles may enhance drug solubility and prolong circulation time and provide passive accumulation in the inflamed kidney tissue due to enhanced permeability and retention (EPR) effects. In addition, their small size and the possibility to modify their surfaces increase uptake by mesangial and tubular epithelial cells, the major targets in nephropathy [34].

Accordingly, the present study aimed to assess the potential beneficial effect of OLM and its OLM-PLGA nanoparticles against MTX-induced nephrotoxicity in male *Sprague–Dawley* rats through AT1/ERK1/2 pathway investigation. Although this model does not fully recapitulate human methotrexate-associated nephrotoxicity, it provides a controlled experimental platform for investigating the mechanisms of renal injury and therapeutic responses with potential relevance to human disease.

## 2. Results

### 2.1. Full Factorial Design Optimal Design Analysis

The Design-Expert^®^ software (Version 13, State-Ease, Minneapolis, MN, USA) determines the optimal experimental model for each response under investigation, ensuring that the highest adjusted R^2^ and the predicted R^2^ are aligned within a deviation of 0.2 [35]. Moreover, additional indicators further corroborated the accuracy and validity of the examined model, including a statistically insignificant lack of fit value alongside an adequate precision metric exceeding 4.0 for all investigated response variables. Table 1 summarizes the regression outcomes corresponding to the examined independent variables, along with the optimal model identified for each response parameter. The compositional details of the OLM-loaded PLGA nanocarrier formulations are outlined in Table 2.

### 2.2. Effect of Design Variables on the Physical Attributes of the OLM-PLGA NPs

#### 2.2.1. Entrapment Efficiency (EE%)

**Figure 2:**
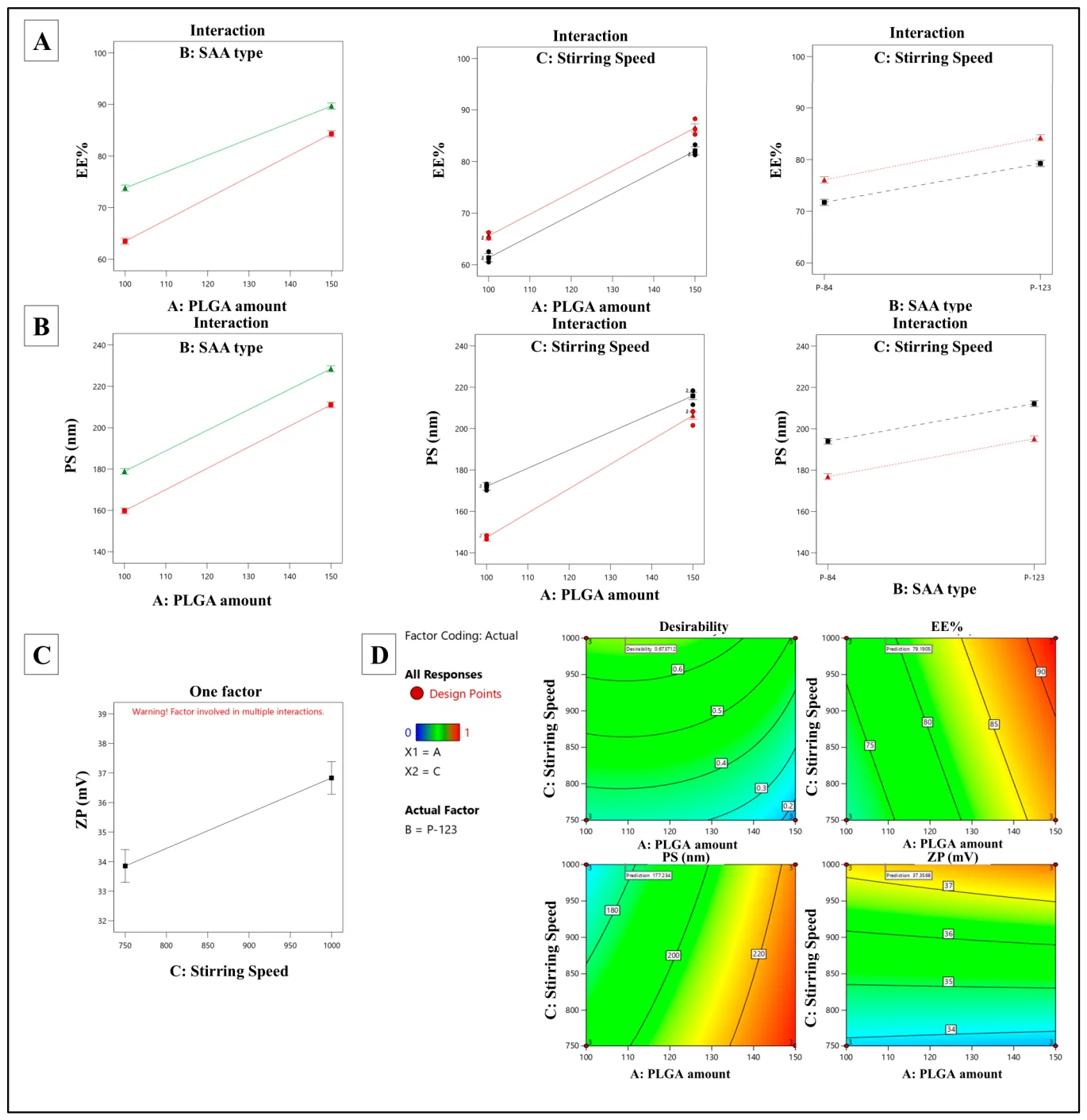
Design-Expert interaction plot for the effect of factors X1 (PLGA amount), X2 (SAA type), and X3 (stirring speed) on design responses, i.e., EE% (**A**), PS (**B**), ZP (**C**), desirability and numerical optimization for OLM-loaded PLGA NPs using the 2^3^ full factorial design (**D**).

The two-factor interaction (2FI) model emerged as the most appropriate mathematical representation for describing the encapsulation efficiency (EE%) response. The statistical parameters associated with this model are comprehensively presented in Table 1, while the experimental EE% values obtained for all formulations are compiled in Table 2. EE% varied from 61.44 ± 1.03 for F1 to 92.12 ± 0.49 for F8. ANOVA indicated that all design variables were significant factors in the model. The quantity of PLGA (X1) was directly related to the encapsulation efficiency percentage (EE%), as seen in Figure 2A. 

This is associated with an increased volume of the polymer matrix in each produced particle, allowing for a greater number of drug molecules to be physically accommodated and encapsulated inside the developing nanoparticle during the nanoprecipitation process. Moreover, an increased quantity of PLGA led to a more viscous organic phase, as previously noted, resulting in expedited polymer precipitation, which may reduce the quantity of the drug that separates into the external aqueous phase [36]. This enhances the quantity of the drug retained by the polymer, thereby elevating the final EE%. Additionally, variations in SAA type (X2) significantly (*p* < 0.001) alter EE%. The augmentation of EE% in nanoparticles synthesized using P-123 compared to those developed with P-84 may be attributed to the comparatively lower HLB value of P-123 relative to P-84 (HLB values: P-123 = 8, P-84 = 14) [37]. Consequently, P-123, exhibiting more hydrophobicity (lower HLB value), would have a superior drug encapsulation efficiency (EE%) with hydrophobic OLM compared to P-84, which has higher HLB values [38]. Moreover, an increase in stirring speed (X3) significantly enhanced the EE% (*p* < 0.0001). In the nanoprecipitation process, nanoparticles are generated by interfacial phenomena, since interfacial turbulence induces convection effects. The solvent movement induced physicochemical instability, resulting in localized areas of supersaturation. Furthermore, the occurrence of turbulence for a certain duration may facilitate the evaporation of the organic solvent and inhibit drug leakage [39].

#### 2.2.2. Particle Size (PS)

All formulations exhibited particle sizes within the nanoscale range (less than 300 nm) (Table 2). All formulation variables (X1, X2, and X3) were significant terms of the model. An increase in PLGA quantity correlated with the expansion of PS (Figure 2B). Elevating the concentration of PLGA augmented particle size due to the increased viscosity of the organic phase. The increased viscosity impedes the diffusion of the organic solvent into the aqueous phase, thus inhibiting the mixing process. This facilitates the aggregation of polymer chains into bigger particles instead of smaller ones [40]. Also, a greater PLGA concentration enhances polymer–polymer interactions, thereby prolonging solvent evaporation and resulting in the creation of bigger particles [40]. Regarding SAA type (X2), the particle size of nanoparticles created with P-123 exhibited a greater size than those produced with P-84. This may be attributable to the difference in HLB values of the two surfactant agents, as previously noted, and the findings were consistent with the particle size data. PS and EE% exhibit a correlation: when EE% rises, the dimensions of the nanoparticles similarly expand [41].

Conversely, the PS exhibited a negative correlation with stirring speed (X3). Elevated stirring velocities generate substantial mechanical and hydraulic shear forces, which are responsible for the formation of nanoparticles possessing diminished average diameters [42]. All the synthesized PLGA nanoparticles exhibited PDI values of 0.15 (Table 2), indicating homogeneity across all formulations and a narrow unimodal distribution curve of particle size.

#### 2.2.3. Zeta Potential (ZP)

The data for ZP is shown in Table 2. The ZP values of all formulations varied from −33.62 ± 1.05 to −37.89 ± 1.15 (absolute value), indicating excellent stability of the synthesized nanoparticles [43]. PLGA nanoparticles generally exhibit a negative zeta potential due to the terminal carboxylic acid groups present in the PLGA chains. The electrostatic repulsion among PLGA nanoparticles increases with the uniform rise in the total number of charged nanoparticles, thus preventing particle aggregation and subsequent size enlargement, ultimately achieving physical stability [44]. In contrast, insufficiently stable nanoparticles (exhibiting lower Zeta potential values) agglomerate, resulting in diminished surface adhesiveness, a distinctive characteristic of nanosized particles that facilitates efficient adherence to biological membranes and enhances absorption [45].

A statistically significant reduction in the mean particle diameter of PLGA nanocarriers was observed in response to elevating the stirring rate (*p* < 0.0001) (Figure 2C), perhaps linked to a heightened absolute zeta potential resulting from an increase in surface area relative to particle volume [46].

### 2.3. Selection and Validation of the Optimized OLM-PLGA NPs

The desirability function was used to select the optimal formulation, with predefined targets for achieving the smallest particle size, the highest zeta potential (absolute value), and the maximum entrapment efficiency. The software suggested the optimal parameters with a desirability value of 0.674, which were: 109.325 mg of PLGA, P-123 as the surfactant, and a stirring rate of 1000 rpm. These conditions were predicted to produce nanoparticles with a particle size of 177.216 nm, an encapsulation efficiency of 79.185%, and a zeta potential of −37.357 mV (absolute value), as shown in Figure 2D. Based on these predictions, the recommended formulation was prepared and subjected to further characterization. High correlation was noticed among the observed and predicted values of optimized OLM-PLGA NPs (Table 3). In addition, the average bias percentages for all obtained responses were smaller than 10%, revealing the model’s high predictive capacity.

[(Predicted value − Observed value)/Observed value] × 100.

#### 2.3.1. In Vitro Release and Release Kinetics Study

**Figure 3:**
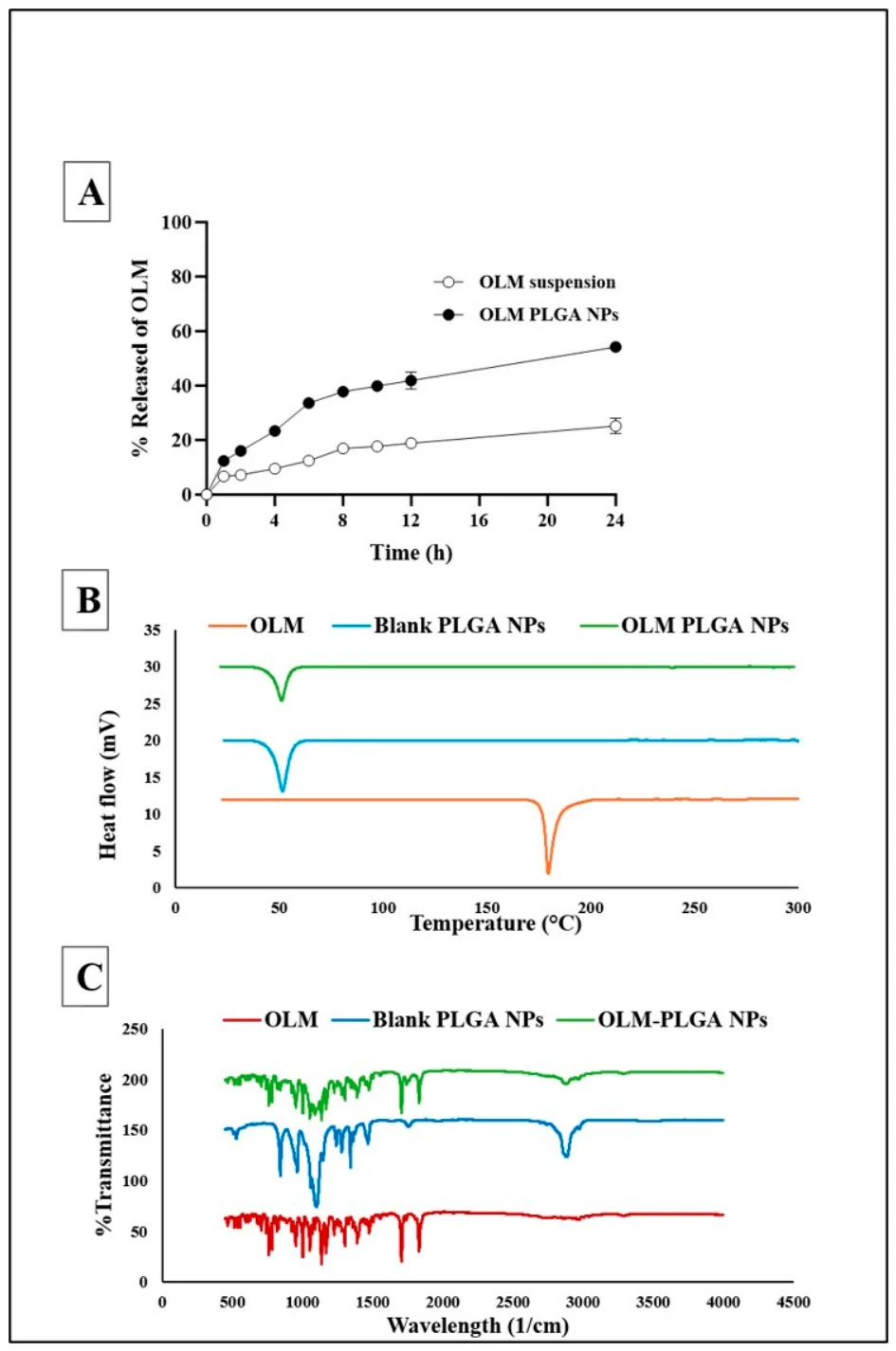
(**A**) Release profile of OLM from PLGA NPs vs. OLM suspension; (**B**) DSC thermogram of pure OLM, blank formulation (drug-free), and optimized OLM-PLGA NPs; (**C**) FTIR spectra of pure OLM, blank formulation (drug-free), and optimized OLM-PLGA NPs.

The in vitro release profiles of OLM from the optimized PLGA nanocarrier dispersion, alongside those of the OLM suspension, are depicted in Figure 3A. 

The nanocarrier formulations exhibited an initial burst release phase, succeeded by a distinctive sustained liberation pattern. Approximately 34% of the encapsulated drug was discharged within the initial 6 h, attributable to the diffusion of surface-adherent and non-encapsulated OLM molecules. This was subsequently followed by a prolonged release phase extending up to 24 h, characterized by a slow and controlled liberation governed by diffusion processes through the intact high-molecular-weight PLGA polymeric matrix [47]. A significant difference was observed between the percent of OLM released from the OLM suspension and those of the OLM-PLGA nanocarrier dispersion. This pronounced enhancement in dissolution rate can be ascribed to the amplified specific surface area resulting from the diminished particle dimensions, in addition to the presence of surfactant moieties. Furthermore, it has been documented that saturation solubility exhibits an inverse relationship with particle size reduction. In accordance with the Noyes–Whitney equation, an elevation in saturation solubility coupled with a decrease in particle diameter collectively contribute to an accelerated dissolution rate. Consequently, formulating poorly water-soluble pharmaceuticals as nanoscale drug particles exerts a substantial influence on dissolution kinetics, drug solubility, and ultimately bioavailability. Given that the bioavailability of OLM is governed by its dissolution rates, reducing the particle dimensions constitutes a promising approach for improving the therapeutic efficacy of the drug.

Kinetic analysis of the release data revealed that the PLGA nanoparticles were most appropriately described by the Korsmeyer–Peppas mathematical model, as evidenced by the highest coefficient of determination (R^2^) values. The calculated release exponent (*n*) was determined to be 0.495, which falls below 0.5, thereby indicating a Fickian diffusion-controlled release mechanism that is concurrently influenced by polymer chain relaxation and erosional processes [48,49,50].

#### 2.3.2. Differential Scanning Calorimetry (DSC)

As seen in Figure 3B, pure OLM had a clear melting peak at 184 °C, which is typical for its crystalline form [51]. On the other hand, the blank nanoparticles (without the drug) did not show any OLM-related peak, with only broad changes related to the amorphous polymer, including the PLGA glass transition temperature between 30 and 60 °C [33]. Interestingly, the OLM-loaded PLGA nanoparticles showed no melting peak for OLM at all, and only polymer-related signals were observed. The disappearance of the OLM melting endotherm may suggest a reduction in OLM crystallinity following its incorporation into the PLGA matrix. This behavior may also be associated with drug encapsulation onto the PLGA surface [52].

#### 2.3.3. Fourier-Transform Infrared (FTIR)

The FTIR spectrum of pure OLM displayed clear characteristic absorption bands at 3285 cm^−1^ (O–H stretching), 2955 cm^−1^ (C–H stretching of aromatic rings), 1765 cm^−1^ (C=O stretching of the carboxylic group), and 1172 cm^−1^ (C–N stretching). These peaks confirmed the identity and purity of the drug, as shown in Figure 3C. The blank PLGA nanoparticles showed typical polymer-related peaks at 1750–1760 cm^−1^ (ester C=O), 1080–1180 cm^−1^ (C–O–C), and 2850–3000 cm^−1^ (aliphatic C–H) [32]. In the OLM-loaded PLGA nanoparticles, the main peaks were those of PLGA, while the OLM-related bands were either weaker or slightly shifted. No new peaks or major changes in the spectrum were observed, indicating the absence of any chemical interaction between OLM and PLGA.

#### 2.3.4. Transmission Electron Microscope (TEM)

**Figure 4:**
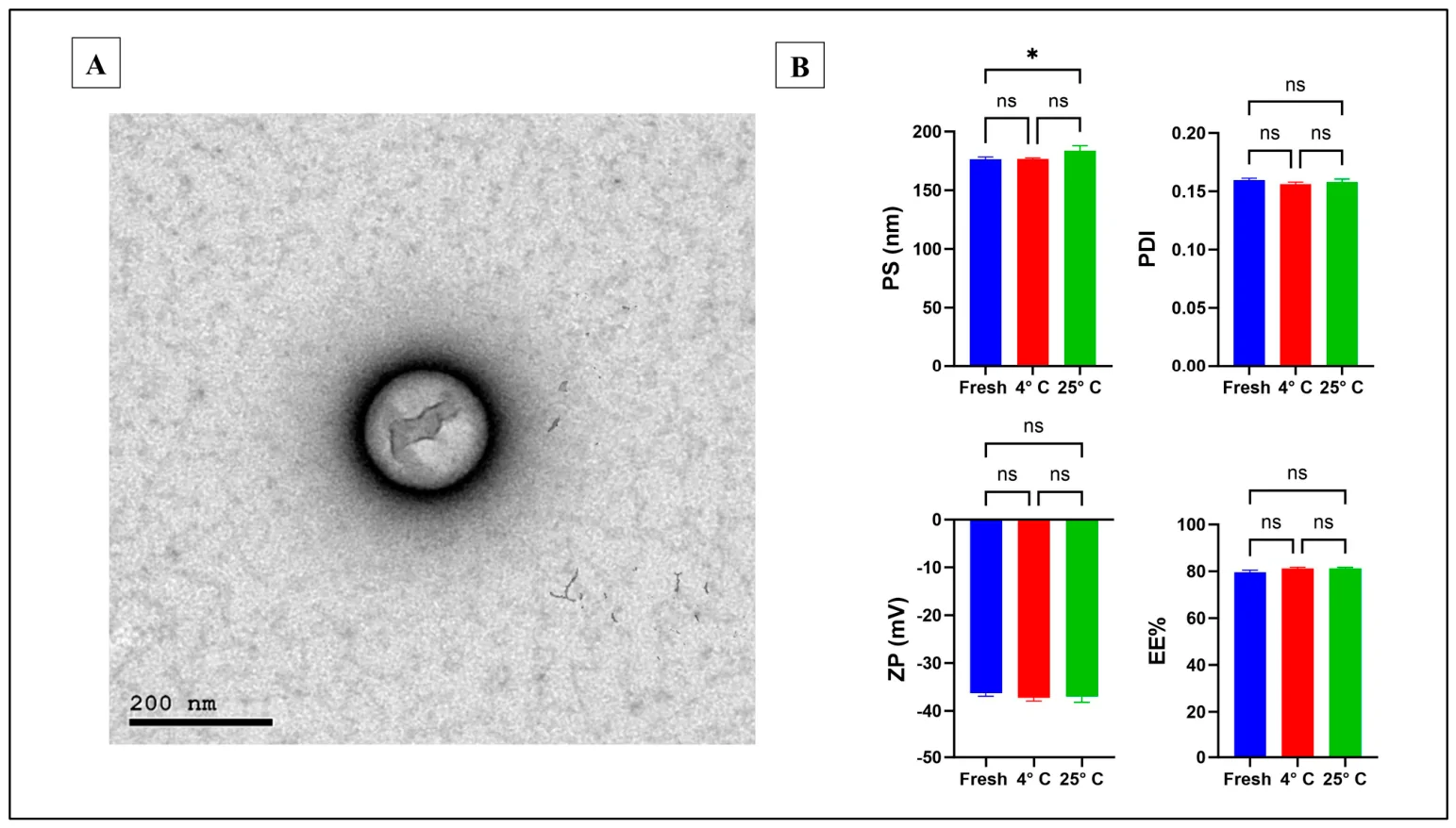
(**A**) TEM image of optimized OLM-PLGA NPs; (**B**) storage stability test results of optimized OLM-PLGANPs on particle size (PS), polydispersity index (PDI), zeta potential (ZP), and entrapment efficiency (EE%) at 4 °C and 25 °C. The data demonstrates a significant increase in particle size during storage, with no significant change in PDI, ZP, and EE% (mean ± SD; * *p* < 0.05; ns, not significant).

TEM micrographs (Figure 4A) validated the effective synthesis and morphological integrity of the produced nanoparticle systems. OLM-PLGA nanoparticles had uniform, spherical morphology with smooth surfaces and a homogenous interior fading core, signifying consistent drug distribution within a solid polymeric matrix. The TEM results confirm effective OLM encapsulation, a maintained nanoscale structure, and a strong correlation between TEM and DLS size measurements.

#### 2.3.5. Stability Study

The stability behavior of the optimized OLM-PLGA nanoparticles is presented in Figure 4B. The formulation maintained good physicochemical stability when kept in a refrigerator at 4 °C, with no significant differences (*p* > 0.05) observed in particle size, PDI, zeta potential, or entrapment efficiency throughout the storage period. In contrast, when stored at 25 °C, a slight but significant increase in particle size (*p* < 0.05) was noticed, indicating reduced stability at this temperature, which is commonly reported for nanoscale drug delivery systems.

### 2.4. In Vivo Nephroprotective Activity

#### 2.4.1. Animal Observations

Throughout the experimental period, the animals were monitored for general health status and potential adverse effects. No expected or unexpected adverse events were observed in any of the experimental groups. No animals were excluded after randomization, and all 24 animals completed the experimental protocol.

#### 2.4.2. Effect of OLM and OLM-PLGA on Body Weight, Kidney Weight, and Relative Kidney Weight Against MTX-Induced Nephrotoxicity in Rats

**Figure 5:**
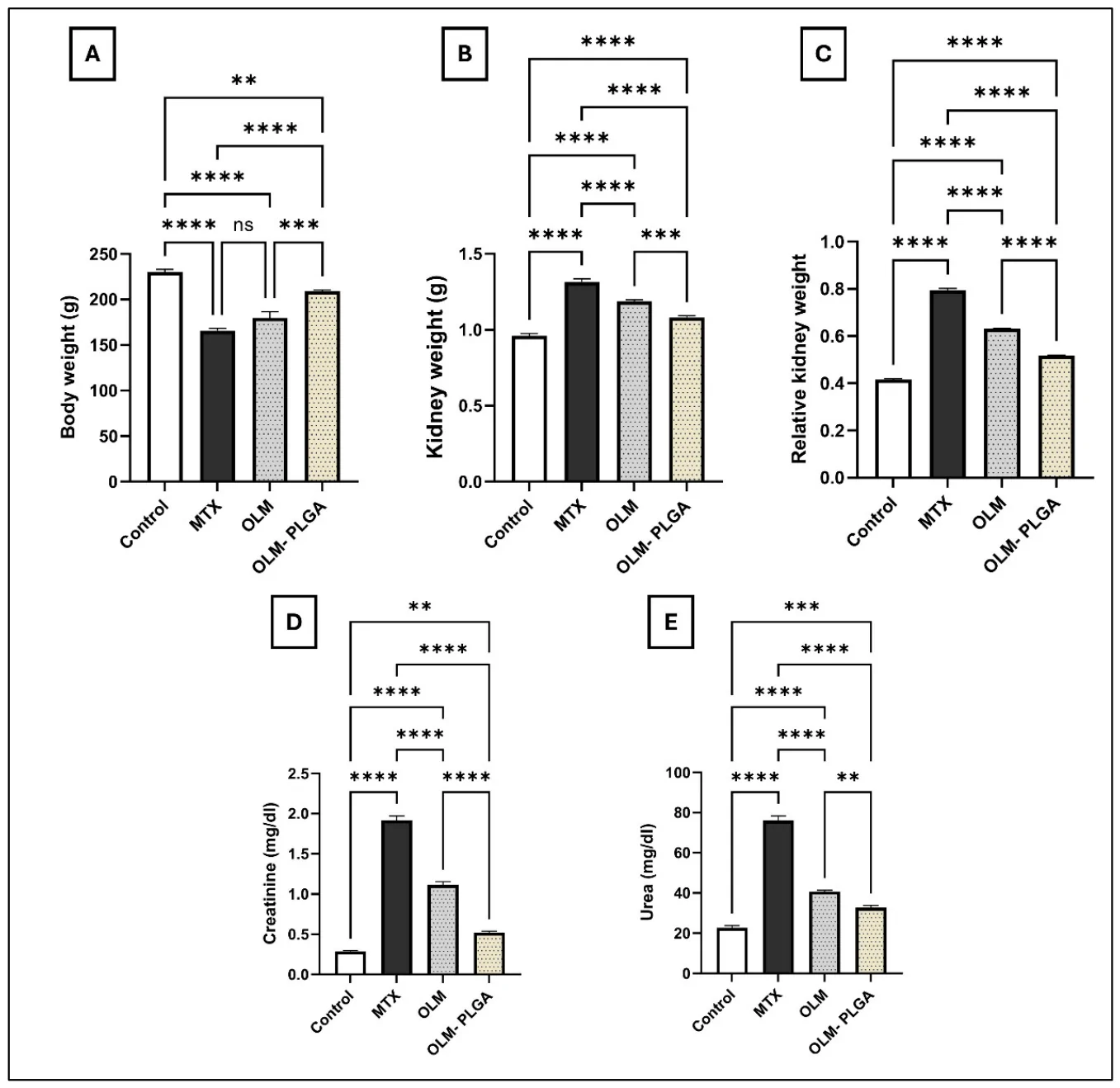
Effect of OLM and OLM-PLGA on (**A**) body weight, (**B**) kidney weight, (**C**) relative kidney weight, and serum kidney function tests of (**D**) Creatinine and (**E**) Urea against MTX-induced nephrotoxicity in rats. Results are expressed as Mean ± SEM (*n* = 6). Statistical significance was conducted through one-way ANOVA, followed by Tukey’s post hoc test, and shown as: ** *p* < 0.01, *** *p* < 0.001, **** *p* < 0.0001; ns = non-significant. MTX: methotrexate, OLM: olmesartan, PLGA: biodegradable poly (lactide-co-glycolic) acid nanoformulation.

MTX administration caused significant renal toxicity, manifested by a 28.0% decrease in body weight (*p* < 0.0001) and significant elevations in kidney weight and relative kidney weight by 1.4-fold and 1.9-fold, respectively, versus the control group (*p* < 0.0001). Both OLM and OLM-PLGA treatments significantly mitigated these changes, reducing kidney weight by 9.6% and 17.7%, respectively, and lowering relative kidney weight by 20.4% and 34.85%, respectively, compared with the MTX group (*p* < 0.0001). Furthermore, rats treated with OLM-PLGA exhibited a significant (*p* < 0.0001) 1.3-fold increase in body weight relative to MTX-treated animals. Superior activity was recorded for OLM-PLGA as it significantly increased body weight by 1.2-fold (*p* < 0.001) and significantly decreased kidney weight and relative kidney weight by 9.0% (*p* < 0.001) and 18.1% (*p* < 0.0001), respectively, in comparison with the OLM-treated group (Figure 5A–C).

#### 2.4.3. Effect of OLM and OLM-PLGA on Kidney Function Tests Against MTX-Induced Nephrotoxicity in Rats

Treatment with MTX caused significant kidney dysfunction and injury, which appeared in the form of an impaired excretory function of the kidney. As a result, the serum levels of both creatinine and BUN were significantly increased by 6.7-fold and 3.4-fold, respectively, upon treatment with MTX (*p* < 0.0001). Meanwhile, treatment with OLM and OLM-PLGA significantly (*p* < 0.05) decreased creatinine by 41.9% and 72.9%, respectively, and urea level by 46.5% and 57.0%, respectively, compared with the MTX-treated group (*p* < 0.0001). Of note, treatment with OLM-PLGA produced a stronger effect than OLM, with a significant reduction in creatinine and BUN by approximately 53.3% (*p* < 0.0001) and 19.7% (*p* < 0.01), respectively, relative to the OLM-treated group (Figure 5D,E).

#### 2.4.4. Effect of OLM and OLM-PLGA on Oxidative Stress Markers Against MTX-Induced Nephrotoxicity in Rats

**Figure 6:**
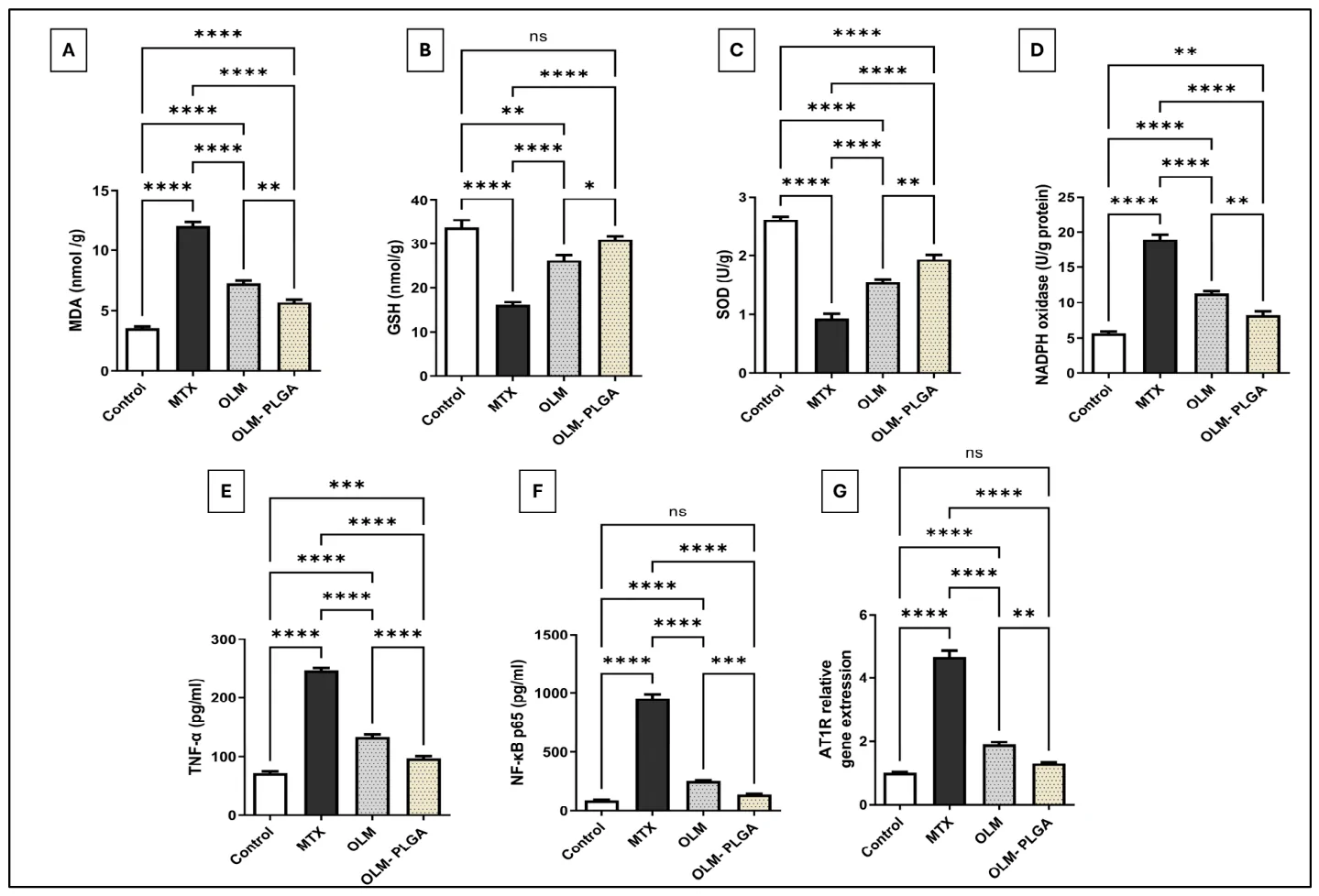
Effect of OLM and OLM-PLGA on oxidative stress markers (**A**) MDA, (**B**) GSH, (**C**) SOD, and (**D**) NADPH; on inflammatory markers (**E**) TNF-α and (**F**) NF-ĸB; and on the gene expression of (**G**) AT1R against MTX-induced nephrotoxicity in rats. Representative cropped Western blot images showing the expression of p-ERK1/2 with β-actin as a loading control. Results are expressed as Mean ± SEM (*n* = 6). Statistical significance was conducted through one-way ANOVA, followed by Tukey’s post hoc test, and shown as: * *p* < 0.05, ** *p* < 0.01, *** *p* < 0.001, **** *p* < 0.0001; ns = non-significant. MTX: methotrexate, OLM: olmesartan, PLGA: biodegradable poly (lactide-co-glycolic) acid nanoformulation, MDA: malondialdehyde, GSH: reduced glutathione, SOD: superoxide dismutase, NADPH oxidase: nicotinamide adenine dinucleotide phosphate (NADPH) oxidase, TNF-α: tumor necrosis factor-alpha, NF-ĸB p65: phosphorylated nuclear factor-kappa B, AT1R: angiotensin II receptor I.

MTX-treated rats exhibited marked oxidative stress, as evidenced by significant (*p <* 0.0001) increases in malondialdehyde (MDA) level and NADPH oxidase activity by approximately 3.4-fold and 3.3-fold, respectively, relative to the control group. In contrast, glutathione (GSH) levels and superoxide dismutase (SOD) activity were significantly (*p* < 0.0001) decreased by approximately 52.2% and 64.3%, respectively, compared with control rats (Figure 6A–D). 

However, treatment with OLM and OLM-PLGA significantly (*p* < 0.0001) decreased MDA level by approximately 39.9% and 52.6%, respectively, and NADPH oxidase activity by approximately 40.3% and 56.4%, respectively. The treatment also increased GSH levels by 1.6-fold and 1.9-fold, respectively, and SOD activity by 1.7-fold and 2.1-fold, respectively, compared to the MTX-treated group. Notably, OLM-PLGA produced more pronounced effects than OLM, showing a significant decline in MDA level and NADPH oxidase activity by approximately 21.1% and 27.1% (*p* < 0.01), together with marked elevations in GSH level and SOD activity by approximately 1.2-fold (*p* < 0.05) and 1.3-fold (*p* < 0.01), respectively, versus the OLM-treated group.

#### 2.4.5. Effect of OLM and OLM-PLGA on Inflammatory Markers Against MTX-Induced Nephrotoxicity in Rats

MTX-treated rats showed a significant (*p* < 0.0001) elevation in the levels of TNF-α and NF-κB p65 by approximately 3.4-fold and 10.4-fold, respectively, compared with the control rats (Figure 6E,F). However, treatment with OLM and OLM-PLGA significantly (*p* < 0.0001) reduced TNF-α levels by approximately 45.9% and 60.7%, respectively, and NF-κB p65 levels by approximately 74.1% and 85.4%, respectively, compared with the MTX-injected rats. OLM-PLGA produced a more significant effect than OLM, showing a significant reduction in TNF-α and NF-κB levels by approximately 27.2% (*p* < 0.0001) and 45.6% (*p* < 0.001), respectively, compared with the OLM-treated group.

#### 2.4.6. Effect of OLM and OLM-PLGA on AT1R Gene Expression Against MTX-Induced Nephrotoxicity in Rats

MTX-injected rats exhibited a significant (*p* < 0.0001) increase in AT1R gene expression by approximately 4.6-fold, relative to the control rats (Figure 6G). However, treatment with OLM and OLM-PLGA significantly (*p* < 0.0001) decreased AT1R gene expression by approximately 59.6% and 72.2%, respectively, compared with the MTX-injected rats. OLM-PLGA showed a more significant effect than OLM, producing a significant reduction in AT1R gene expression by approximately 31.3% (*p* < 0.01), relative to the OLM-treated group.

#### 2.4.7. Effect of OLM and OLM-PLGA on Total and p-ERK1/2 Protein Expression Against MTX-Induced Nephrotoxicity in Rats

**Figure 7:**
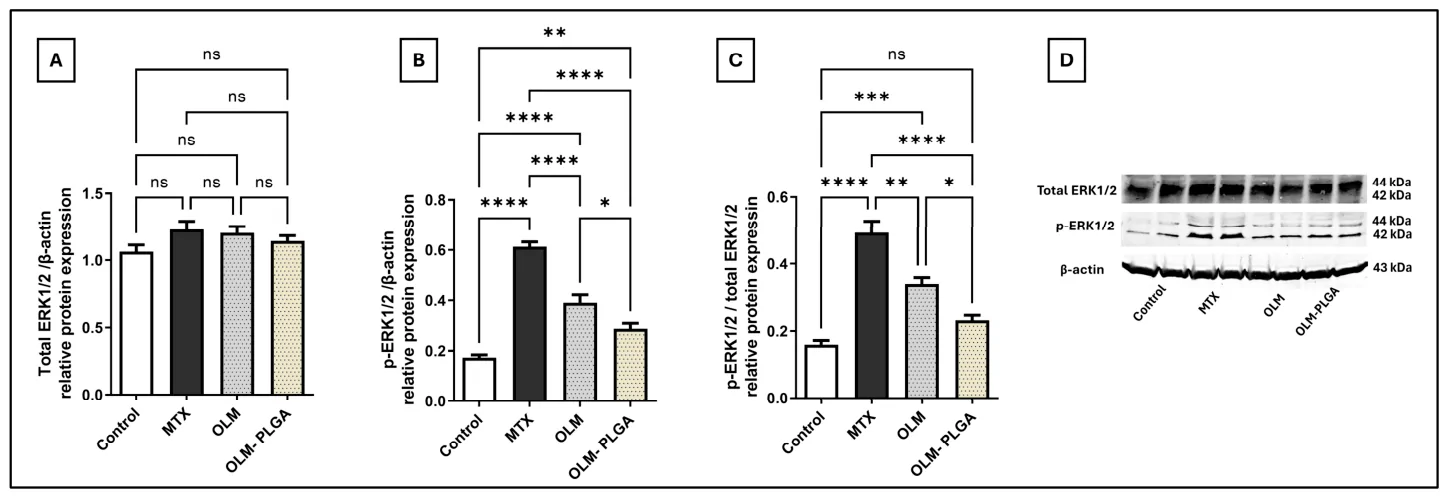
Effect of OLM and OLM-PLGA on the protein expression of (**A**) total ERK1/2, (**B**) p-ERK1/2, and (**C**) p-ERK1/2/total ERK1/2 ratio against MTX-induced nephrotoxicity in rats. (**D**) Representative cropped Western blot images showing the expression of total and p-ERK1/2, with β-actin as a loading control. Results are expressed as Mean ± SEM (*n* = 6). Statistical significance was conducted through one-way ANOVA, followed by Tukey’s post hoc test, and shown as: * *p* < 0.05, ** *p* < 0.01, *** *p* < 0.001, **** *p* < 0.0001; ns = non-significant. MTX: methotrexate, OLM: olmesartan, PLGA: biodegradable poly (lactide-co-glycolic) acid nanoformulation. ERK1/2: extracellular signal-regulated kinase1/2, p-ERK1/2: phosphorylated extracellular signal-regulated kinase1/2.

The relative protein expression of total ERK1/2 exhibited a non-significant change among all groups. Meanwhile, the MTX-treated group exhibited a significant (*p* < 0.0001) increase in p-ERK1/2 protein expression and the relative p-ERK1/2/total ERK1/2 ratio by approximately 3.5-fold and 3.1-fold, respectively, compared to the control rats (Figure 7A–D, and Supplementary Materials). In contrast, administration of OLM and OLM-PLGA significantly (*p* < 0.0001) reduced p-ERK1/2 protein expression by approximately 36.2% and 53.1%, respectively, and reduced the relative p-ERL1/2/total ERK1/2 ratio by 31.1% and 52.9%, respectively, compared with the MTX-injected group. Moreover, OLM-PLGA demonstrated a greater effect than OLM, as evidenced by a significant decline in p-ERK1/2 protein expression and relative p-ERL1/2/total ERK1/2 ratio by approximately 26.4% and 31.6%, respectively, compared with the OLM-treated group.

#### 2.4.8. Effect of OLM and OLM-PLGA on Histopathological Examinations Against MTX-Induced Nephrotoxicity in Rats

**Figure 8:**
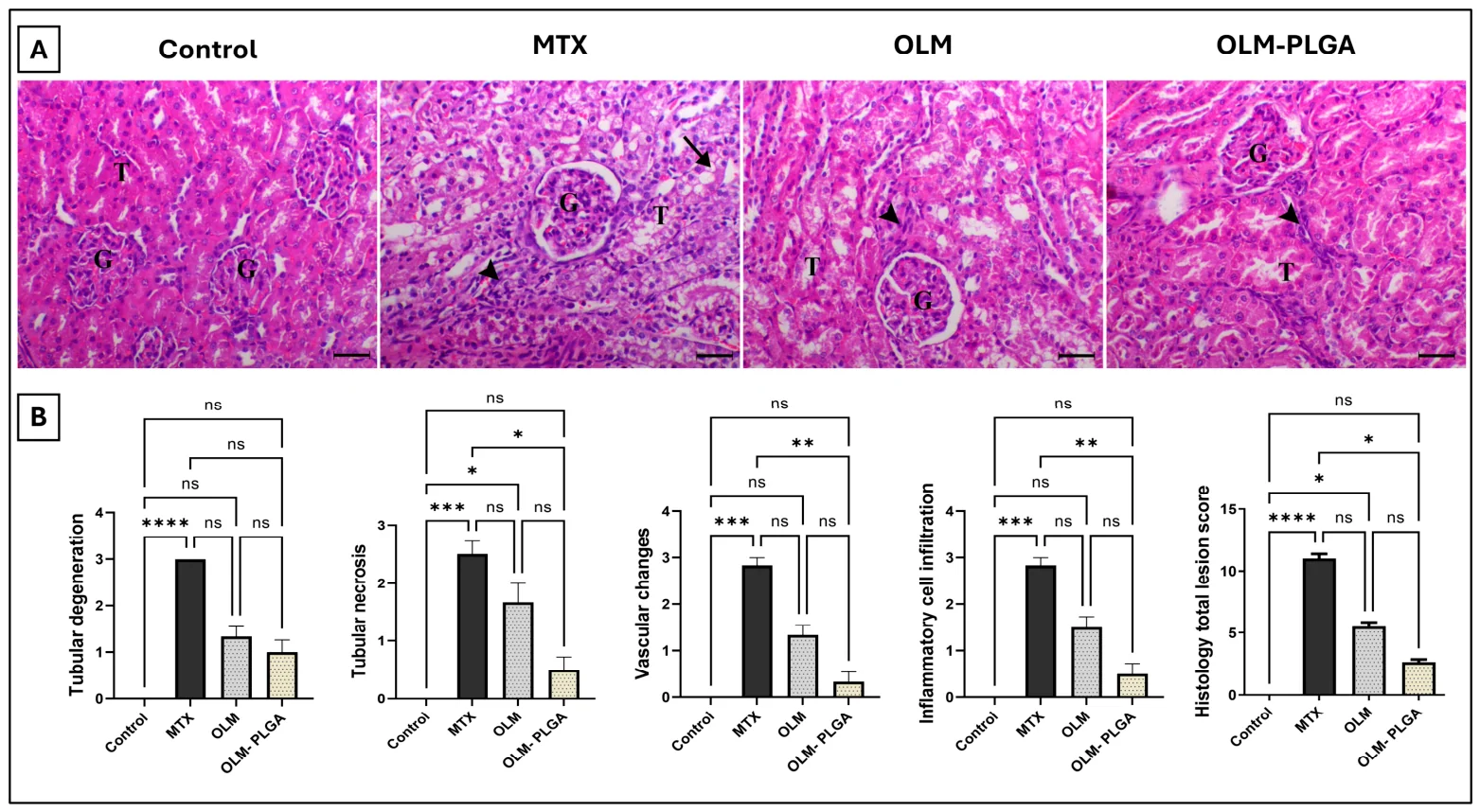
Effect of OLM and OLM-PLGA on (**A**) histopathological examinations and (**B**) histological lesion score, against MTX-induced nephrotoxicity in rats. Histopathological examination of kidney sections revealed normal renal architecture in the control group, with intact glomeruli (G) and tubules (T). In contrast, the MTX group demonstrated severe tubular degenerative changes, with marked vacuolation of the renal tubular epithelium (arrow) and remarkable fibroblastic cell proliferation (arrowhead), while G and T indicated the renal glomeruli and renal tubules, respectively. Treatment with OLM resulted in mild renal tubular eosinophilic degeneration accompanied by mild fibroblastic proliferation (arrowhead), with preservation of most renal structures. Meanwhile, treatment with OLM-PLGA markedly ameliorated the renal histopathological alterations, showing a marked decrease in renal degenerative changes within the renal tubules along with mild fibroblastic proliferation (arrowhead). H&E stain, X200, bar= 50 µm. Histopathological scoring of total renal lesions was represented as Mean ± SEM (*n* = 6) and analyzed nonparametrically via the Kruskal–Wallis test, followed by Dunn’s multiple comparison test. Significance is shown as: * *p* < 0.05, ** *p* < 0.01, *** *p* < 0.001, **** *p* < 0.0001; ns = non-significant. MTX: methotrexate, OLM: olmesartan, PLGA: biodegradable poly (lactide-co-glycolic) acid nanoformulation.

Histopathological examination of renal sections in the control group revealed intact kidney architecture and normal histological features, with intact glomeruli (G) and tubules (T). In contrast, the MTX group demonstrated severe tubular degenerative changes, with marked vacuolation of the renal tubular epithelium (arrow) and remarkable fibroblastic cell proliferation (arrowhead), while G and T indicated the renal glomeruli and renal tubules, respectively. Treatment with OLM resulted in mild renal tubular eosinophilic degeneration accompanied by mild fibroblastic proliferation (arrowhead), with the preservation of most renal structures. Meanwhile, treatment with OLM-PLGA markedly ameliorated the renal histopathological alterations, showing a marked decrease in renal degenerative changes within the renal tubules along with mild fibroblastic proliferation (arrowhead) (Figure 8A). The histological total lesion score was significantly (*p* < 0.0001) increased in the MTX group by approximately 11-fold, compared with the control rats (Figure 8B). However, treatment with OLM and OLM-PLGA significantly (*p* < 0.05) inhibited the total lesion score by approximately 50.0% and 75.6%, respectively, compared with the MTX-injected rats. OLM-PLGA significantly decreased the total lesion score by approximately 51.5%, compared with the OLM-treated group.

#### 2.4.9. Effect of OLM and OLM-PLGA on Apoptotic Markers Against MTX-Induced Nephrotoxicity in Rats

**Figure 9:**
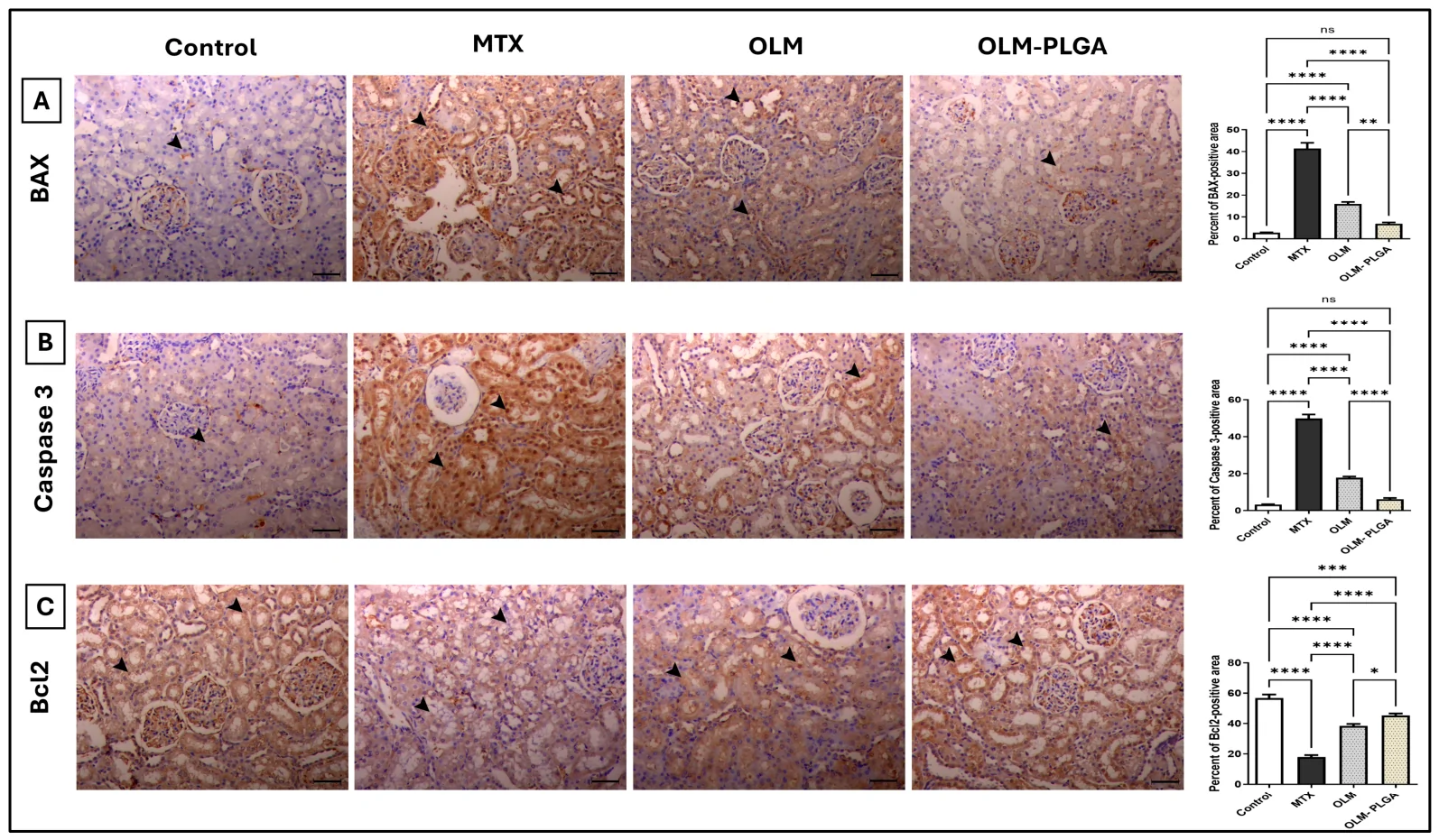
Effect of OLM and OLM-PLGA on apoptotic markers: (**A**) BAX, (**B**) Caspase-3, and (**C**) Bcl-2, against MTX-induced nephrotoxicity in rats. Results are expressed as Mean ± SEM (*n* = 6). Statistical significance was conducted through one-way ANOVA, followed by Tukey’s post hoc test, and shown as: * *p* < 0.05, ** *p* < 0.01, *** *p* < 0.001, **** *p* < 0.0001; ns = non-significant. MTX: methotrexate, OLM: olmesartan, PLGA: biodegradable poly (lactide-co-glycolic) acid nanoformulation.

MTX-treated group showed a significant (*p* < 0.0001) elevation in the levels of Bax (Figure 9A) and caspase-3 (Figure 9B) by approximately 10.4-fold and 15.2-fold, respectively, while significantly (*p* < 0.0001) decreasing the Bcl-2 level (Figure 9C) by approximately 68.2%, compared with the control rats. However, treatment with OLM and OLM-PLGA significantly (*p* < 0.0001) decreased Bax levels by approximately 73.1% and 85.4%, respectively, and caspase-3 levels by approximately 64.3% and 87.7 %, respectively, while significantly increasing the Bcl-2 level by approximately 2.2-fold and 2.5-fold, respectively, compared with the MTX-injected rats. OLM-PLGA showed a significant anti-apoptotic effect compared with OLM, with a significant reduction in Bax and caspase-3 levels by approximately 45.6% (*p* < 0.01) and 65.5% (*p* < 0.0001), respectively, together with a marked elevation in Bcl-2 level by approximately 1.2-fold (*p* < 0.05), compared with the OLM-treated group.

#### 2.4.10. Effect of OLM and OLM-PLGA on α-SMA Expression Against MTX-Induced Nephrotoxicity in Rats

**Figure 10:**
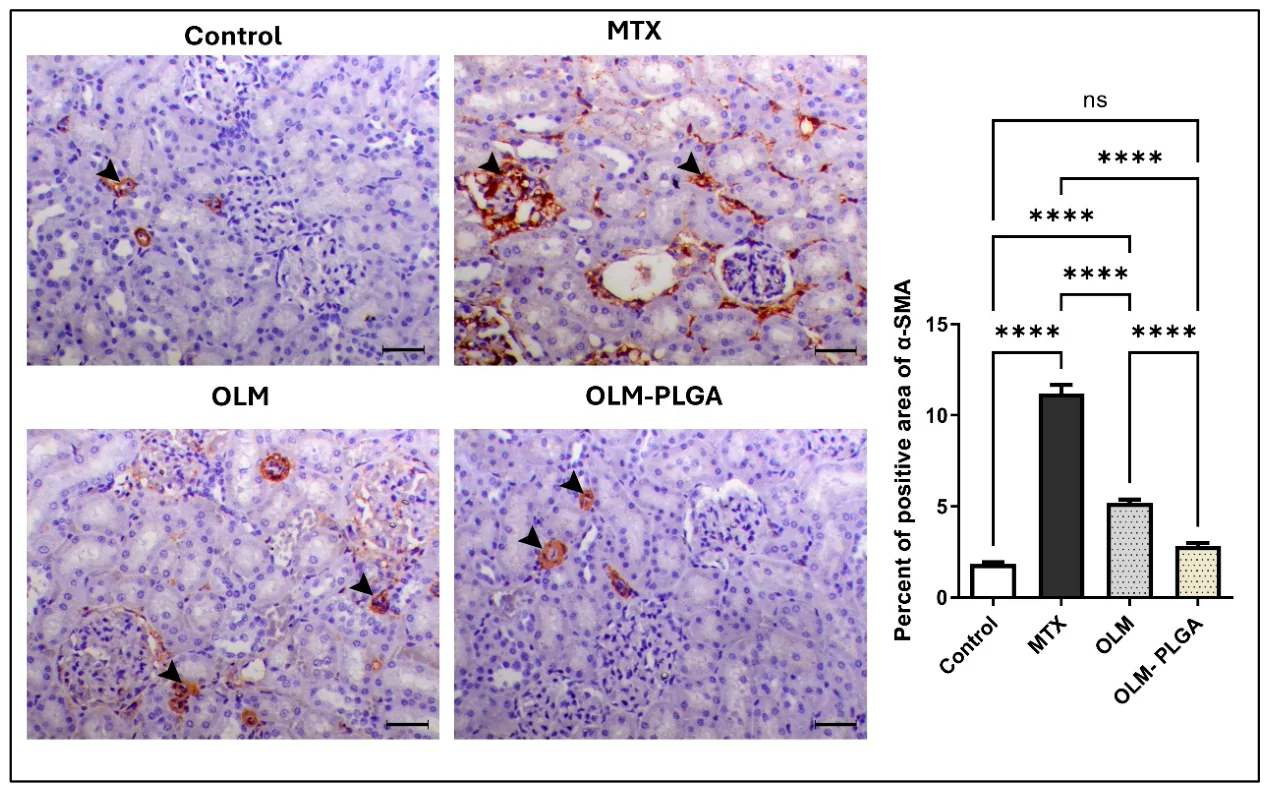
Effect of OLM and OLM-PLGA on α-SMA expression against MTX-induced nephrotoxicity in rats. Kidney of the normal control animal showing mild expression of the α-SMA antibody within the blood vessels of the interstitial tissues (arrowhead). Meanwhile, samples of the MTX-treated group showed marked immunostaining of glomerular and interstitial expression of the α-SMA antibody (arrowheads). Samples from OLM-treated rats showed a decrease in α-SMA immunostaining within the renal glomerular and interstitial tissues (arrowheads), and a further reduction in α-SMA immunoexpression was found in samples from OLM-PLGA-treated rats. Results are expressed as Mean ± SEM (*n* = 6). Statistical significance was conducted through one-way ANOVA, followed by Tukey’s post hoc test, and shown as: **** *p* < 0.0001; ns = non-significant. MTX: methotrexate, OLM: olmesartan, PLGA: biodegradable poly (lactide-co-glycolic) acid nanoformulation, α-SMA: alpha-smooth muscle actin.

The expression of α-SMA was significantly (*p* < 0.0001) increased in rats injected with MTX by approximately 6.1-fold, compared with the control rats (Figure 10). However, treatment with OLM and OLM-PLGA significantly (*p* < 0.0001) decreased α-SMA expression by approximately 53.7% and 74.7%, respectively, compared with the MTX-injected rats. OLM-PLGA significantly decreased α-SMA expression by approximately 45.4% (*p* < 0.0001), compared with the OLM-treated group.

#### 2.4.11. Effect of OLM and OLM-PLGA on Collagen Content Against MTX-Induced Nephrotoxicity in Rats

**Figure 11:**
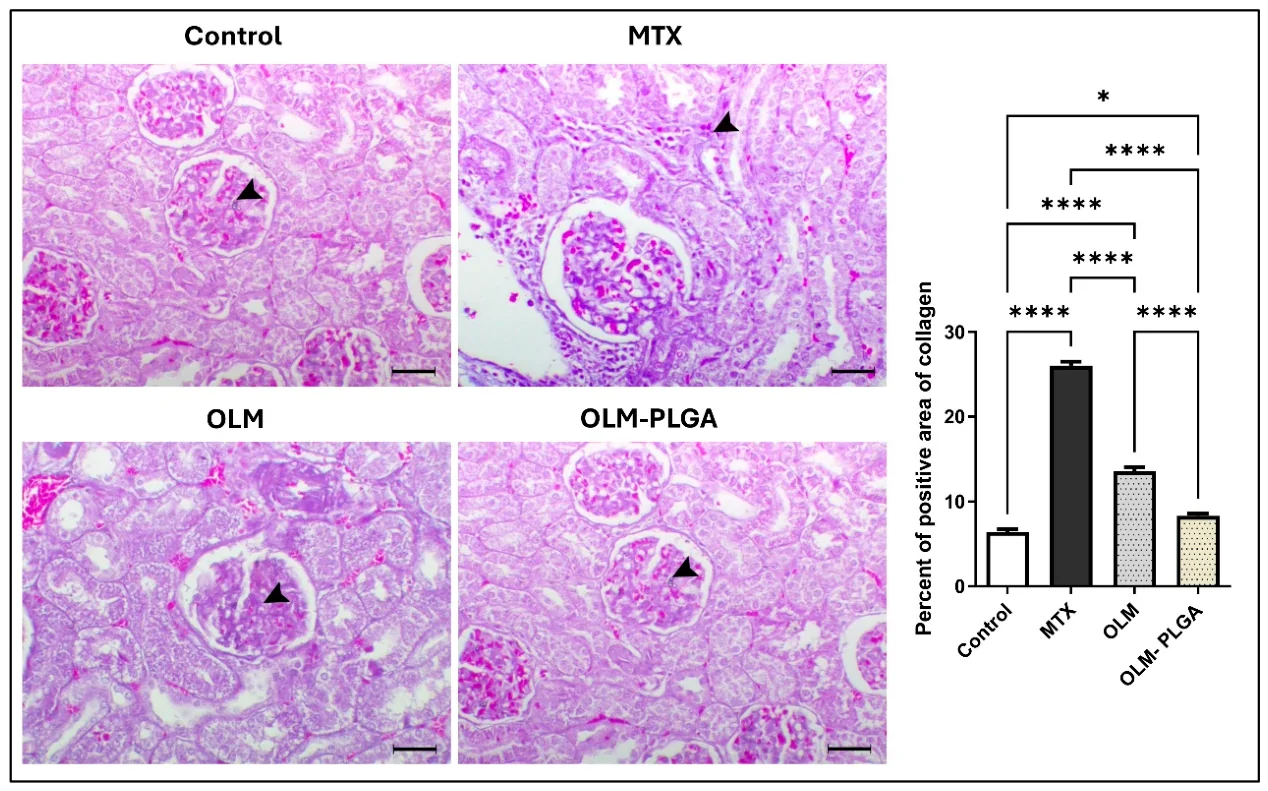
Effect of OLM and OLM-PLGA on collagen content against MTX-induced nephrotoxicity in rats. Masson’s trichrome-stained sections of the control group revealed only mild glomerular and interstitial fibrous connective tissue stroma (arrowhead). However, the MTX-treated group exhibited marked periglomerular and interstitial fibrous connective tissue proliferation (arrowhead). Administration of OLM reduced these fibrotic changes, showing only a mild increase in glomerular fibrous connective tissue (arrowhead). Similarly, OLM-PLGA treatment demonstrated mild glomerular and interstitial fibrous connective tissue within the stroma (arrowhead), indicating attenuation of renal fibrosis compared with the MTX-treated group. Masson’s trichrome stain, X200, bar= 50 µm. Results are expressed as Mean ± SEM (*n* = 6). Statistical significance was conducted through one-way ANOVA, followed by Tukey’s post hoc test, and shown as: * *p* < 0.05, **** *p* < 0.0001. MTX: methotrexate, OLM: olmesartan, PLGA: biodegradable poly (lactide-co-glycolic) acid nanoformulation.

Masson’s trichrome-stained sections of the MTX-treated group showed that collagen content was significantly (*p* < 0.0001) elevated in rats treated with MTX by approximately 4.1-fold, compared with the control rats, with marked periglomerular and interstitial fibrous connective tissue proliferation (Figure 11). However, treatment with OLM and OLM-PLGA significantly (*p* < 0.0001) decreased collagen content by approximately 47.8% and 68.1%, respectively, compared with the MTX-injected rats. OLM-PLGA produced a more significant effect than OLM, showing a significant reduction of approximately 38.9% (*p* < 0.0001), compared with the OLM-treated group.

## 3. Discussion

In the present study, MTX showed marked renal injury characterized by oxidative stress, inflammation, apoptosis, and fibrotic remodeling. OLM and OLM-PLGA significantly attenuated these alterations, indicating a nephroprotective effect. The present findings suggest that modulation of the AT1R/ERK1/2 signaling axis may contribute to the protective effects of OLM and OLM-PLGA, owing to the fact that OLM is an AT1R blocker.

A central finding of the current study is the downregulation of ERK1/2 phosphorylation following OLM and OLM-PLGA treatments. Angiotensin II signaling through AT1R is a well-established activator of the ERK pathway, which regulates inflammation, cell proliferation, and fibrogenesis [17,53]. In the present study, MTX-induced renal damage was associated with increased p-ERK1/2 expression and an increased p-ERK1/2/total ERK1/2 ratio, suggesting enhanced ERK1/2 phosphorylation during renal injury. Importantly, total ERK1/2 protein expression did not differ significantly among the experimental groups, indicating that the observed changes in p-ERK1/2 were not attributable to alterations in total ERK1/2 protein abundance. This observation is consistent with earlier findings demonstrating that AT1R activation promotes ERK1/2 phosphorylation in animal models, contributing to organ damage and fibrosis progression [54,55]. However, OLM and OLM-PLGA significantly reduced p-ERK1/2 expression and the p-ERK1/2/total ERK1/2 ratio, indicating attenuation of ERK1/2 phosphorylation. In parallel, both treatments reduced AT1R mRNA expression. Given the established relationship between AT1R signaling and ERK1/2 activation, these findings are consistent with modulation of the AT1R/ERK1/2 signaling axis during the nephroprotective response. Therefore, the reduction in p-ERK1/2 expression observed in the treated groups provides a key mechanistic link between AT1R inhibition and renal protection. The superior efficacy observed with OLM-PLGA nanoparticles may be related to formulation-associated improvements in OLM dissolution and sustained release, as suggested by the in vitro release profile.

ERK1/2 signaling has a pivotal role in renal fibrogenesis via promoting fibroblast activation and downstream extracellular matrix accumulation [17]. In the current study, MTX administration significantly induced α-SMA immunohistochemical expression and collagen deposition, indicating activation of myofibroblasts and progression of renal fibrosis. The antifibrotic efficacy of OLM and OLM-PLGA was demonstrated by a marked reduction in both α-SMA expression and collagen content. These findings are consistent with involvement of the AT1R/ERK1/2 signaling axis in the modulation of renal fibrotic responses. Therefore, the AT1R///ERK1/2 axis represents a main mechanistic pathway through which OLM reduces renal fibrosis in MTX-induced nephrotoxicity. These findings are in agreement with published data demonstrating that blockade of angiotensin II signaling reduces renal fibrosis by inhibiting MAPK/ERK-mediated fibroblast activation and collagen production [56].

Beyond fibrosis, ERK1/2 inhibition also contributes to modulating oxidative stress [57]. In the present study, MTX significantly increased MDA levels and NADPH oxidase activity while decreasing the GSH level, indicating severe oxidative stress, in agreement with the study of Mohyeldin et al. (2026) [58]. Notably, treatment with both OLM and OLM-PLGA significantly reversed these pathological changes. This antioxidant efficacy may be associated with downstream of AT1R blockade, given that angiotensin II signaling conventionally activates NADPH oxidase through both ERK-dependent and non-ERK-dependent pathways. Consequently, the observed suppression of p-ERK1/2 expression is likely associated with increased NADPH oxidase activity, leading to attenuated reactive oxygen species (ROS) production and the subsequent restoration of cellular redox homeostasis. These findings are consistent with reports showing that OLM reduces oxidative stress through inhibition of ROS generation [21,22].

Inflammatory signaling is heavily dependent upon the activation of the ERK1/2 cascade [59]. In the current study, MTX markedly elevated NF-κB p65 and TNF-α levels, supporting activation of inflammatory pathways. OLM and OLM-PLGA markedly suppressed these inflammatory markers. This anti-inflammatory effect could mechanistically be linked to decreased p-ERK1/2 expression, as ERK signaling can enhance NF-κB activation and cytokine production [59,60]. The suppression of NF-κB observed following OLM and OLM-PLGA treatment was accompanied by reduced AT1R gene expression and ERK1/2 phosphorylation, suggesting that modulation of the AT1R/ERK1/2 axis may contribute to the observed anti-inflammatory response [22,23].

Our results indicated that MTX administration induced significant apoptosis, as evidenced by elevated Bax and caspase-3 immunohistochemical expression and declined Bcl-2 immunohistochemical expression. These findings are similar to those in a previous study of Arab et al. (2022) [61]. OLM and OLM-PLGA treatment significantly decreased the expression of these apoptotic markers in agreement with previous reports documenting the anti-apoptotic potential of OLM [24,25]. The observed attenuation of apoptosis was accompanied by modulation of the AT1R/ERK1/2 signaling axis, suggesting that this pathway may contribute to the anti-apoptotic response. Specifically, the alleviation of cellular inflammation and oxidative stress preserves mitochondrial integrity, which in turn can prevent the activation of intrinsic apoptotic cascades.

Considering the continued clinical use of MTX and the absence of established pharmacological strategies to prevent its nephrotoxicity, enhancing the therapeutic efficacy of an already approved antihypertensive agent through nanotechnology may represent a feasible translational approach. The present findings support that OLM exerts nephroprotective effects against MTX-nephrotoxicity, primarily through an AT1 receptor blockade, inhibition of p-ERK1/2 expression, attenuation of NADPH oxidase activity, reduction in redox disruption, suppression of NF-κB-mediated inflammation, inhibition of apoptosis, suppression of a-SMA expression, and collagen deposition. These interconnected effects collectively may contribute to the preservation of renal structure and function in MTX-induced nephrotoxicity.

## 4. Materials and Methods

### 4.1. Drugs and Chemicals

Olmesartan medoxomil (OLM) was supplied by FAP Pharmaceutical Co. (Cairo, Egypt). Acid-terminated poly(lactic-co-glycolic acid) (PLGA 75:25) (Purasorb PDLG 7502A; 75:25 DL-lactide/glycolide copolymer; molecular weight 66,000–107,000 Da) was purchased from Corbion Purac (Gorinchem, The Netherlands). Pluronic P-123 (P-123) and Pluronic F-84 (F-84) were obtained from BASF (Seoul, Republic of Korea). A cellulose dialysis membrane (molecular weight cut-off of 12–14 kDa) was sourced from SERVA Electrophoresis (Heidelberg, Germany). Ethanol (95%) and acetone were supplied by El-Nasr Pharmaceutical Chemicals Co. (Cairo, Egypt). Methotrexate (MTX) was provided by Hikma Pharmaceutical Co. (6th of October City, Egypt) in the form of vials containing 50 mg/2 mL.

### 4.2. Preparation of Olmesartan-Polylactic-Co-Glycolic Acid (OLM-PLGA) Nanoformulation

#### 4.2.1. Experimental Design

A 2^3^ full factorial design was employed to evaluate the effects of formulation variables on the physicochemical characteristics of PLGA nanoparticles using Design-Expert^®^ Software version 13 (Stat-Ease Inc., Minneapolis, MN, USA). The study included eight experimental runs, with entrapment efficiency (EE%, Y1), particle size (PS, Y2), and zeta potential (ZP, Y3) selected as the dependent responses. The independent variables were PLGA concentration (X1), surfactant type (X2), and stirring speed (X3), as presented in Table 1. Analysis of variance (ANOVA) was performed to determine the statistical significance of the factors investigated on the measured responses. The composition of the prepared nanoparticle formulations and the corresponding response values are summarized in Table 2 and expressed as mean ± SD (*n* = 3).

#### 4.2.2. Assembly of OLM-PLGA Nanoparticles

OLM-encapsulated PLGA nanoparticles were fabricated using the nanoprecipitation approach [52]. Briefly, 20 mg of OLM and a predetermined quantity of PLGA were co-dissolved in 5 mL of an acetone–methanol mixture (60:40 *v*/*v*) using ultrasonication (Crest Ultrasonics Corp., Ewing Township, NJ, USA) for one minute. The resultant organic phase was then added dropwise into 10 mL of an aqueous surfactant mixture (containing 1% *w*/*v* P-84 or P-123) under continuous magnetic stirring at 1500 rpm and ambient temperature. The volatile organic solvents were subsequently eliminated by evaporation at room temperature under mechanical agitation at 1500 rpm for one hour. To further reduce particle dimensions, the obtained colloidal suspensions were subjected to an additional ultrasonication step for three minutes [31]. The final nanoparticle dispersions were stored under refrigeration until further analysis.

### 4.3. Characterization of the Assembled OLM-PLGA Nanoparticles

#### 4.3.1. Particle Size (PS), Size Distribution (PDI), and Zeta Potential (ZP)

The developed PLGA nanoparticle suspensions were characterized with respect to the average hydrodynamic diameter, polydispersity index (PDI), and zeta potential using a Zetasizer Nano ZS-90 apparatus (Malvern Instruments, Southboro, MA, USA). Prior to analysis, each specimen was diluted 15-fold with double-distilled water and examined at a fixed scattering angle of 90° under thermostatically controlled conditions (25 °C) [62].

#### 4.3.2. Entrapment Efficiency (EE%)

The encapsulation efficiency (%) of OLM within the PLGA nanocarriers was established employing a direct assay method. Briefly, the nanoparticle dispersions were ultracentrifuged at 20,000 rpm for 60 min at 4 °C using a Hermle Z326K centrifuge (Hermle Labortechnik GmbH, Wehingen, Germany). The pelleted nanoparticles were subsequently lysed with acetonitrile, and the liberated OLM content was determined spectrophotometrically at its characteristic absorption maximum of 258 nm utilizing a UV–Vis spectrophotometer (Shimadzu, Tokyo, Japan). The percentage entrapment efficiency was computed in accordance with the equation reported by [63]:

$$EE\%=\frac{Entraped\;amount\;of\;OLM\;in\;nanoparticles}{Total\;amount\;of\;OLM\;in\;formulation’s\;dispersion}\times 100$$

#### 4.3.3. Selection and Validation of the Optimized OLM-PLGA Nanoparticles

The identification of the most favorable OLM-PLGA nanoparticle composition was accomplished through the application of a desirability function, guided by predefined criteria that prioritized minimizing the average particle diameter while concurrently maximizing both the absolute zeta potential value and the drug encapsulation efficiency. The nanocarrier system exhibiting the highest desirability index was subsequently fabricated, thoroughly characterized, and its experimental outcomes were compared against the predicted values generated by Design-Expert^®^ Software version 13 (Stat-Ease Inc., Minneapolis, MN, USA) [62].

#### 4.3.4. Differential Scanning Calorimetry (DSC)

Thermal behavior investigations were performed on pure OLM, the freeze-dried optimized OLM-loaded PLGA nanoparticles, and the lyophilized blank PLGA nanocarriers employing a DSC-50 calorimeter (Shimadzu, Kyoto, Japan). Each specimen was positioned within an aluminum pan and subjected to a controlled temperature elevation from 25 °C to 300 °C at a constant heating rate of 10 °C/min under a protective nitrogen atmosphere [31].

#### 4.3.5. Fourier-Transform Infrared (FT-IR)

The FT-IR spectral characterization was executed on pure OLM, the lyophilized optimized OLM-loaded PLGA nanoparticles, and the lyophilized blank PLGA nanoparticles utilizing a Vertex80 FT-IR spectrometer (Bruker, Berlin, Germany). All spectra were acquired across the wavenumber range of 400–4000 cm^−1^ at ambient temperature (25 °C) [64].

#### 4.3.6. In Vitro Release and Release Kinetic Studies

The release study was conducted using the United States Pharmacopeia (USP) dissolving equipment (Pharma Test, Hainburg, Germany) for a duration of 24 h at 37 °C. One-milliliter samples (containing 2 mg of OLM) from the formulated dispersions were inserted in plastic cylinder tubes, with one end securely sealed with a cellulose, while the other end was affixed to the shaft of the USP dissolving apparatus instead of the baskets. The formulations were submerged in a 50 mL combination of PBS (pH 7.4) and ethanol (30:20, *v*/*v*) [65]. The sink condition was preserved in this volume. Aliquots were extracted at 1, 2, 4, 6, 8, 12 and 24 h. OLM in aliquots was examined using a UV spectrophotometer at λmax 258 nm. The experiment was conducted in triplicate. The OLM release mechanism was investigated by using several mathematical models to the in vitro release data, including the zero-order, first-order, Higuchi, and Korsmeyer–Peppas models [66].

#### 4.3.7. Transmission Electron Microscopy (TEM)

The morphological characteristics and dimensional attributes of the optimized PLGA nanocarrier formulation in its colloidal state were examined utilizing transmission electron microscopy (TEM) (JEOL RI 2100, JEOL, Ltd., Freising, Germany). A single drop of the nanoparticle dispersion was appropriately diluted, negatively stained with an aqueous solution of 0.1% (*w*/*v*) phosphotungstic acid, and subsequently deposited onto a carbon-coated copper grid affixed to a specimen holder. Micrographs were captured at varying magnification levels to thoroughly visualize the nanoparticles [66].

### 4.4. Stability Study on OLM-PLGA NPs

The optimized OLM-loaded PLGA nanoparticle samples were subjected to a storage stability investigation over a six-month period under two distinct temperature conditions, 4 °C and 25 °C. At predefined intervals, the stored specimens were visually inspected for any observable signs of physical instability, including droplet coalescence, particle sedimentation, and alterations in coloration. Formulation stability was assessed by comparing the initial and 6-month measurements of particle size, polydispersity index (PDI), zeta potential, and entrapment efficiency (EE%) [66].

### 4.5. Assessment of In Vivo Nephroprotective Activity

#### 4.5.1. Animals

Twenty-four adult male *Sprague–Dawley* rats (180–200 g) were obtained from the breeding facility of the Egyptian Organization of Biological Products and Vaccines (Helwan, Egypt). The animals were housed for a one-week acclimatization period prior to experimentation under controlled environmental conditions, including a temperature of 25 ± 2 °C and a 12 h light/dark cycle. Rats were provided with a standard diet and water *ad libitum* throughout the study. Animals were included only if they exhibited normal behavior with no signs of disease or injury. Animals meeting these criteria were randomly assigned to the four experimental groups (*n* = 6 per group). Random allocation was performed by serially drawing individual rats from the holding cage and assigning them to one of the four treatment groups using a lottery method (i.e., each rat was placed into a cage chosen at random without replacement until all groups reached *n* = 6).

Animals showing signs of illness, injury, significant weight loss, or abnormal behavior before study initiation or during the study period were excluded. Any deviations from the approved experimental protocol, including incorrect dosing or improper sample collection, result in exclusion from final analysis.

The experiment was held in the animal house at the Faculty of Pharmacy, Capital University. Animals were handled in accordance with the guidelines for animal care, which were approved by the Institutional Animal Care and Use Committee, Capital University, Faculty of Pharmacy (ethical protocol number: IACUC 23A 2025).

#### 4.5.2. Experimental Design

Twenty-four adult male Sprague–Dawley rats were randomly assigned into four groups (*n* = 6 per group).

- Group 1 (Control): Rats received 10% Tween 80 in normal saline (1 mL/kg, p.o.) for 14 consecutive days as a vehicle.
- Group 2: MTX-treated group: Rats were given a single intraperitoneal injection of methotrexate (20 mg/kg) on day 1 [67,68]. One hour post-administration, they received 10% Tween 80 in normal saline (1 mL/kg, p.o.) daily for 14 days.
- Group 3: OLM-treated group: Animals received a single intraperitoneal dose of MTX (20 mg/kg) on day 1, followed one hour later by oral administration of OLM (10 mg/kg, p.o.) for 14 days [69].
- Group 4: OLM-PLGA-treated group: Animals received a single intraperitoneal dose of MTX (20 mg/kg) on day 1, followed one hour later by oral administration of OLM-PLGA (10 mg/kg, p.o.) for 14 days.

Free OLM and OLM-PLGA were administered at a nominal OLM dose of 10 mg/kg. For OLM-PLGA, the required dose volume was calculated according to the initial OLM concentration used during formulation. The unentrapped OLM fraction was not separated from the nanoparticle dispersion prior to administration.

Animals were monitored throughout the experimental period for general health status and any signs of treatment-related adverse effects.

Humane endpoints were predefined and included severe lethargy, inability to access food or water, persistent recumbency, or body weight loss exceeding 20% of baseline. Animals reaching these criteria were to be humanely euthanized and excluded from further analysis. No animals met the predefined humane endpoint criteria during the study.

All animals were maintained under identical experimental conditions. Potential confounders were addressed by standardizing handling, treatment administration, and measurement timing across all groups.

The authors responsible for administering treatments were not blinded to group allocation due to the nature of the treatment. However, blinding was implemented during outcome assessment and data analysis. The investigator evaluating outcomes (body weight, kidney weight, biochemical assays, histopathological examination) was blinded to group allocation, and all samples were coded before analysis to ensure unbiased interpretation.

#### 4.5.3. Body Weight Monitoring

The body weights of the rats were monitored periodically throughout the experimental period. The recorded body weights were used primarily to adjust the administered doses of OLM and OLM-PLGA according to the current body weight of each animal. The final body weights were recorded before sacrifice and subsequently analyzed as an experimental outcome and used to assess treatment-related changes and calculate relative kidney weight.

#### 4.5.4. Blood Sampling and Tissue Collection

At the end of the study, rats were anesthetized with sodium thiopental (40 mg/kg, I.P.) [70], and blood samples were collected via retro-orbital puncture using heparinized capillary tubes. The blood was allowed to clot at room temperature for 30 min, then centrifuged at 3000 rpm for 15 min. The resulting clear serum was separated for renal function assessment. After that, rats were sacrificed, and both kidneys were isolated, washed with phosphate-buffered saline, dried, and weighed to calculate the relative kidney weight. The right-kidney samples were stored in 10% neutral-buffered formalin for histopathological examination. Left-kidney tissues were homogenized in 0.1 M phosphate-buffered saline (PBS, pH 7.4). The resulting homogenates were centrifuged at 10,000 rpm for 30 min at 4 °C, and the supernatants were collected and stored at −80 °C for subsequent biochemical analyses.

#### 4.5.5. Determination of Body Weight, Kidney Weight and Relative Kidney Weight

For each animal, body and kidney weights were recorded, and relative kidney weight was calculated using the following equation [71]:

Relative kidney weight (%) = (kidney weight (g)/body weight (g)) × 100

#### 4.5.6. Assessment of Kidney Function Tests (BUN and Creatinine)

BioVision colorimetric assay kits were used to measure BUN and creatinine levels (Cat # K376-100 and K625-100, respectively; BioVision, Inc., Milpitas, CA, USA). All assays were performed in accordance with the manufacturer’s instructions.

#### 4.5.7. Assessment of Oxidative Stress Markers

Oxidative stress biomarkers, including MDA, GSH, and SOD, were measured using commercial assay kits (Cat. # MD 25 29, GR 25 11, and SD 25 21, respectively; Bio-Diagnostic, Dokki, Giza, Egypt). All steps were carried out in accordance with the manufacturer’s instructions. Meanwhile, NADPH oxidase was determined using Elabscience NADPH Oxidase (NOX) assay kit (Cat # E-BC-K815-M, Elabscience Biotechnology Inc., Houston, TX, USA).

#### 4.5.8. Assessment of TNF-α and NF-κB p65

Inflammatory biomarkers, including TNF-α and NF-κB p65, were quantified using commercially available ELISA kits (Cat # SL0722Ra; SunLong Biotech Co., Hangzhou, China, and Cat # E-EL-R0674; Elabscience Biotechnology Inc., Houston, TX, USA, respectively). All procedures were performed in accordance with the manufacturer’s instructions.

#### 4.5.9. Assessment of AT1R Expression by Real Time-PCR Methods

RNA Total RNA was isolated from renal tissues using the Qiagen RNeasy^®^ Plus Mini Kit (Cat. No. 74134, Hilden, Germany). RNA concentration and purity were assessed using a NanoDrop ND-1000 spectrophotometer (NanoDrop Technologies, Wilmington, Wilmington, DE, USA) based on absorbance at 260 nm and the 260/280 nm ratio. Complementary DNA (cDNA) was synthesized from RNA samples using a High-Capacity RNA-to-cDNA Master Mix kit. Quantitative PCR amplification was carried out using gene-specific primers (Table 4), which were designed via Primer-BLAST (Primer 3, version 2.5.0), and performed with Thermo Scientific Maxima SYBR Green/ROX qPCR Master Mix (2X) on a Rotor-Gene Q system (Qiagen, Germantown, MD, USA). Relative gene expression levels were calculated from threshold cycle (Ct) values using the 2^−ΔΔCt^ method.

#### 4.5.10. Assessment of Phosphorylated and Total ERK 1/2 Protein Expression by Western Blot Analysis

Total protein was extracted from renal tissues using the ReadyPrep™ Total Protein Extraction Kit (Cat. No. 163–2086, Bio-Rad Laboratories, Hercules, CA, USA) following homogenization. Protein concentration was measured using the Bradford Protein Assay Kit (Cat. No. SK3041, BioBasic Inc., Markham, ON, Canada). Subsequently, proteins were separated by SDS-PAGE and transferred onto membranes for immunoblot analysis. The membranes were incubated with the primary antibodies against total ERK1/2 (Cat. No. A4782, ABclonal Biotech Co., Wuhan, China) and p-ERK1/2 (Cat. No. 4370, Cell Signaling Technology, Danvers, MA, USA), followed by incubation with horseradish peroxidase (HRP)-conjugated secondary antibodies. Protein detection was performed using Clarity™ Western ECL substrate (Cat. No. 170–5060, Bio-Rad Laboratories, Hercules, CA, USA), and chemiluminescent signals were visualized using a CCD-based imaging system. Densitometric analysis of protein bands was carried out using ChemiDoc MP software (version 3, Bio-Rad Laboratories Inc., Hercules, CA, USA), with β-actin used as the internal loading control for normalization.

#### 4.5.11. Histopathological Assessment

For histopathological examination, kidney tissues were thoroughly perfused and fixed in 10% neutral-buffered formalin for 72 h. After fixation, samples were processed through trimming, dehydration in a graded ethanol series, clearing with xylene, and embedding in a Paraplast paraffin medium. Serial sections of 5 μm thickness were obtained using a rotary microtome for evaluation of renal histoarchitecture. The sections were stained with hematoxylin and eosin (H&E) and examined under a light microscope for morphological assessment. Histopathological changes were evaluated and scored depending on the following criteria—tubular degeneration, necrosis, vascular changes including congestion and hemorrhage, and inflammatory cell infiltration—on a scale of 0 to 3 (0: none, 1: mild, 2: moderate, and 3: severe) for each criterion, followed by summation of the whole criteria with a 12-point score. To estimate collagen content, Masson’s trichrome stain was used on sections. Six representative non-overlapping fields were randomly determined for both histopathological scoring and quantification of staining intensity using ImageJ software (version 2.0, NIH, Bethesda, MA, USA), and representative images were captured for analysis.

#### 4.5.12. Assessment of BAX, Bcl-2, Caspase-3, and α-SMA by Immunohistochemical Investigation

Paraffin-embedded sections were deparaffinized and subjected to antigen retrieval by immersion in 0.05 M citrate buffer (pH 6.8). Thereafter, endogenous peroxidase activity was quenched using 0.3% H_2_O_2_, followed by incubation with a protein-blocking solution. The sections were then incubated with primary antibodies, including Bax monoclonal antibody (Invitrogen, Rockford, IL, USA, Cat. No. MA5-14003, 1:200), polyclonal anti-caspase-3 antibody (Invitrogen, Cat. No. PA5-77887, 1:100 dilution), Bcl-2 polyclonal antibody (Invitrogen, Cat. No. PA5-27094, 1:200 dilution), and α-SMA monoclonal antibody (Thermo Fisher, Rockford, IL, USA, Cat. No. MA5-11547, 1:800 dilution). After washing with phosphate-buffered saline (PBS), Bax-stained sections were incubated with a mouse secondary monoclonal antibody (Cat. No. K3468, EnVision+™ System Horseradish Peroxidase Labelled Polymer; Dako), whereas the remaining antibodies were incubated with a goat anti-rabbit secondary antibody (Cat. No. K4003, EnVision+™ System Horseradish Peroxidase Labelled Polymer; Dako) for 30 min at room temperature. Immunoreactivity was visualized using a DAB chromogen kit and counterstained with Mayer’s hematoxylin. Staining intensity was quantified as the percentage of positively stained area within a total area of 1 mm^2^ using ImageJ software (NIH, Bethesda, MA, USA).

### 4.6. Statistical Analysis

Data from the preparation and characterization of the OLM–PLGA nanoformulation were expressed as mean (M) ± standard deviation (SD), whereas results from the in vivo experiments were presented as mean (M) ± standard error of the mean (SEM). Data distribution was assessed for normality using the Shapiro–Wilk test. Statistical comparisons among experimental groups were performed using one-way analysis of variance (ANOVA) followed by Tukey’s post hoc multiple comparison test. Histopathological scoring data were analyzed using the Kruskal–Wallis test, followed by Dunn’s post hoc multiple comparison test. All statistical analyses were conducted using GraphPad Prism software (version 9; GraphPad Software, Inc., San Diego, CA, USA). A *p*-value of less than 0.05 was considered statistically significant.

## 5. Study Limitation

While the results of this study show potential, there are several important concerns that need to be addressed before these findings can be applied in clinical settings. Firstly, the model only reflects an acute chemical injury, which does not adequately mimic the chronic effect of MTX human nephrotoxicity. Secondly, the study focused solely on male rats, which may not fully reflect sex- or species-specific responses.

No formal a priori sample-size or power calculation was performed before the study. Although the sample size was selected based on the experimental design and relevant previous studies, the absence of a prospective power analysis limits the ability to formally determine whether the study was adequately powered to detect all potentially relevant differences. Future studies should incorporate a priori power calculations based on the expected effect sizes and variability of the primary outcomes to provide a more rigorous justification of sample size.

Moreover, the modulation of AT1R/ERK1/2 axis pathways was assessed based on biochemical and molecular assessments, but causal relationships were not confirmed using pathway-specific inhibitors or genetic inhibition/activation approaches.

Although the optimized OLM-PLGA formulation demonstrated greater nephroprotective effects than free OLM, the lack of an MTX + blank PLGA group prevents the contribution of the PLGA carrier itself from being independently excluded or quantified. Similarly, inclusion of a healthy + blank PLGA group would have provided additional information regarding any baseline effects of the nanoparticle carrier.

The quantitative stability assessment was based on initial and 6-month measurements; therefore, intermediate changes in formulation characteristics could not be characterized.

Furthermore, pharmacokinetic and biodistribution studies were not performed; thus, the effects of PLGA nanoencapsulation on systemic OLM exposure, circulation time, tissue distribution, and renal accumulation could not be directly established. Consequently, the observed superiority of OLM-PLGA over free OLM cannot be conclusively attributed to enhanced systemic bioavailability or renal delivery. Future studies incorporating plasma pharmacokinetic profiling and tissue biodistribution analysis are warranted to determine whether altered OLM exposure and renal accumulation contribute to the enhanced nephroprotective effect.

Finally, the experimental protocol evaluated the nephroprotective effects of OLM and OLM-PLGA over a relatively short treatment period and does not address the long-term efficacy, safety, pharmacokinetics, or pharmacological behavior of the nanoformulation. Longer-term studies incorporating pharmacokinetic and biodistribution assessments are required.

## 6. Conclusions

The present findings demonstrate that OLM confers significant protection against methotrexate-induced renal injury, an effect that was markedly enhanced following PLGA nanoencapsulation. Both free OLM and OLM-loaded PLGA nanoparticles preserved renal function and improved histological architecture and were associated with favorable changes in oxidative stress-related, inflammatory, apoptotic, and fibrotic markers. These nephroprotective effects were associated with the modulation of the AT1R/ERK1/2 signaling axis, as evidenced by reduced AT1R gene expression and ERK1/2 phosphorylation, together with suppression of NADPH oxidase activity, reductions in NF-κB p65 and TNF-α levels, modulation of apoptosis-related mediators, and reduced α-SMA expression and collagen accumulation. The superior nephroprotective effects observed with OLM-PLGA may be associated with the formulation’s physicochemical characteristics and sustained-release behavior observed in vitro. Collectively, these findings support the potential of OLM-PLGA nanoparticles as a promising nanotherapeutic strategy for the prevention of MTX-associated nephrotoxicity and warrant further pharmacokinetic, biodistribution, and mechanistic studies to support its future application.

## Figures and Tables

**Table 1:** The 2^3^ full factorial design summary and statistics used for OLM-PLGA nanoparticle optimization.

Independent Variables (Factors)	Constraints		
X1: PLGA amount (mg)	100	150	
X2: SAA type	P-84	P-123	
X3: Stirring speed (rpm)	750	1000	
Dependent variables (Responses)	Constraints		
Y1:EE%	Maximize		
Y2:PS (nm)	Minimize		
Y3: ZP (absolute value)	Maximize		
**Responses**	**Y1: EE%**	**Y2: PS (nm)**	**Y3: ZP**
Minimum	60.52	146.56	−32.56
Maximum	92.45	235.25	−38.56
Ratio	1.53	1.61	1.18
Model	2FI	2FI	2FI
Adequate precision	57.484	68.764	8.596
R^2^	0.9935	0.9953	0.8370
Adjusted R^2^	0.9912	0.9937	0.7795
Predicted R^2^	0.9870	0.9907	0.6751
Model *p*-value	<0.0001	<0.0001	<0.0001
Model F-Value	431.41	605.33	14.55
Significant variables	X1, X2, X3	X1, X2, X3	X3

Abbreviations: 2FI: Two-factor interactions; OLM: olmesartan; PLGA: polylactic co-glycolic acid; SAA: surface active agent; P-84: pluronic F-84; P-123: pluronic P-123; PS; particle size; EE%: the percentage of entrapment efficiency; ZP: zeta potential.

**Table 2:** Output data of the 2^3^ full factorial design for OLM-PLGA nanoparticles.

Formulations Factor				Responses (Mean ± SD)			
Formulation Code	X1 PLGA Amount (mg)	X2 SAA Type	X3 Stirring Speed	Y1 EE%	Y2 PS (nm)	Y3 ZP (mV)	PDI
F1	100	P-84	750	61.44 ± 1.03	171.88 ± 1.58	−33.89 ± 0.66	0.153 ± 0.001
F2	100	P-123	1000	71.28 ± 1.2	191.41 ± 0.91	−33.86 ± 1.47	0.155 ± 0.004
F3	100	P-84	750	65.55 ± 0.61	147.69 ± 0.98	−36.5 ± 0.65	0.156 ± 0.002
F4	100	P-123	1000	76.28 ± 1.16	166.32 ± 1.68	−37.22 ± 0.95	0.157 ± 0.003
F5	150	P-84	750	81.98 ± 1.1	216.06 ± 3.89	−33.82 ± 0.64	0.155 ± 0.001
F6	150	P-123	1000	87.19 ± 0.55	232.92 ± 2.08	−33.62 ± 1.05	0.153 ± 0.001
F7	150	P-84	750	86.58 ± 1.53	206.06 ± 3.89	−37.16 ± 0.53	0.156 ± 0.001
F8	150	P-123	1000	92.12 ± 0.49	224.02 ± 1.67	−37.89 ± 1.15	0.153 ± 0.001

Abbreviations: OLM: olmesartan; PLGA: polylactic co-glycolic acid, SAA: surface active agent; P-84: pluronic F-84; P-123: pluronic P-123; PS: particle size; EE%: the percentage of entrapment efficiency; ZP: zeta potential; PDI: polydispersity index. Data are shown as mean ± SD (*n* =3). F1–F8 corresponded to the eight distinct compositions tested according to the 2^3^ full factorial design.

**Table 3:** The observed, predicted values and bias percent of optimized OLM-PLGA NPs.

	Formulations Factor			Responses		
Formulation Code	X1 PLGA Amount (mg)	X2 SAA Type	X3 Stirring Speed	Y1 EE%	Y2 PS (nm)	Y3 ZP (mV)
Observed Values of Optimized OLM-PLGA NPs	109.325	P-123	1000	79.56	176.33	−36.30
Predicted Values of Optimized OLM-PLGA NPs	109.325	P-123	1000	79.18	177.21	−37.35
Bias *				−0.47	0.49	2.89

Abbreviations: OLM: olmesartan; PLGA: polylactic co-glycolic acid; SAA: surface active agent; P-123: pluronic P-123; PS: particle size; EE%: the percentage of entrapment efficiency; ZP: zeta potential. * Bias % calculated from this equation:

**Table 4:** Primers used in the study.

Parameter	Primer Sequence (5′→ 3′)	Accession Number
AT1R	F-TTCGTGGCTTGAGTCCTGTT R-ATCCACTTGACCAGGGAATG	XM_008771594.4
β-actin	F- AGGAGTACGATGAGTCCGGC R-CGCAGCTCAGTAACAGTCCG	NM_031144.3

## Data Availability

The original contributions presented in this study are included in the article/Supplementary Materials. Further inquiries can be directed to the corresponding author.

## Supplementary Materials

The following supporting information can be downloaded at: https://www.mdpi.com/article/10.3390/ph19091462/s1, Uncropped image of Western blot analysis of p-ERK ½ and β-actin.

## Abbreviations

The following abbreviations are used in this manuscript:

ANOVA	Analysis of variance
AT1R	Angiotensin II type 1 receptor
Bax	Bcl-2-associated X protein
Bcl-2	B-cell lymphoma 2
BUN	Blood urea nitrogen
cDNA	Complementary deoxyribonucleic acid
Ct	Cycle threshold
DSC	Differential scanning calorimetry
EE%	Entrapment efficiency percentage
ECL	Enhanced chemiluminescence
ELISA	Enzyme-linked immunosorbent assay
ERK1/2	Extracellular signal-regulated kinase 1/2
FTIR	Fourier-transform infrared spectroscopy
GSH	Glutathione
H&E	Hematoxylin and eosin
HRP	Horseradish peroxidase
MDA	Malondialdehyde
MTX	Methotrexate
NADPH	Nicotinamide adenine dinucleotide phosphate
NF-κB	Nuclear factor-kappa B
NPs	Nanoparticles
OLM	Olmesartan
OLM-PLGA	Olmesartan-loaded poly(lactic-co-glycolic acid) nanoparticles
PBS	Phosphate-buffered saline
PDI	Polydispersity index
PLGA	Poly(lactic-co-glycolic acid)
PS	Particle size
qPCR	Quantitative polymerase chain reaction
ROS	Reactive oxygen species
SAA	Surface active agent
SD	Standard deviation
SDS-PAGE	Sodium dodecyl sulfate–polyacrylamide gel electrophoresis
SEM	Standard error of the mean
SOD	Superoxide dismutase
TEM	Transmission electron microscopy
TNF-α	Tumor necrosis factor-alpha
ZP	Zeta potential
