# Supplementary material for: Olmesartan-Loaded PLGA Nanoparticles Attenuate Methotrexate-Induced Kidney Injury: Association with the AT1R/ERK1/2 Signaling Axis
Source: Pharmaceuticals (Basel). 2026 Sep 15;19(9):1462. doi: 10.3390/ph19091462 (PMC13610287; doi:10.3390/ph19091462)
Supplement: Supplementary file 1 [file pharmaceuticals-19-01462-s001.zip › pharmaceuticals-4525213-supplementary.pdf]

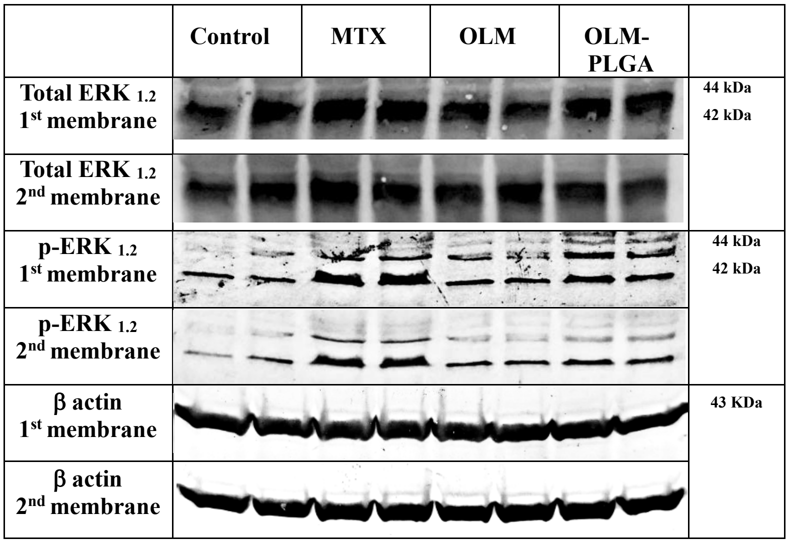

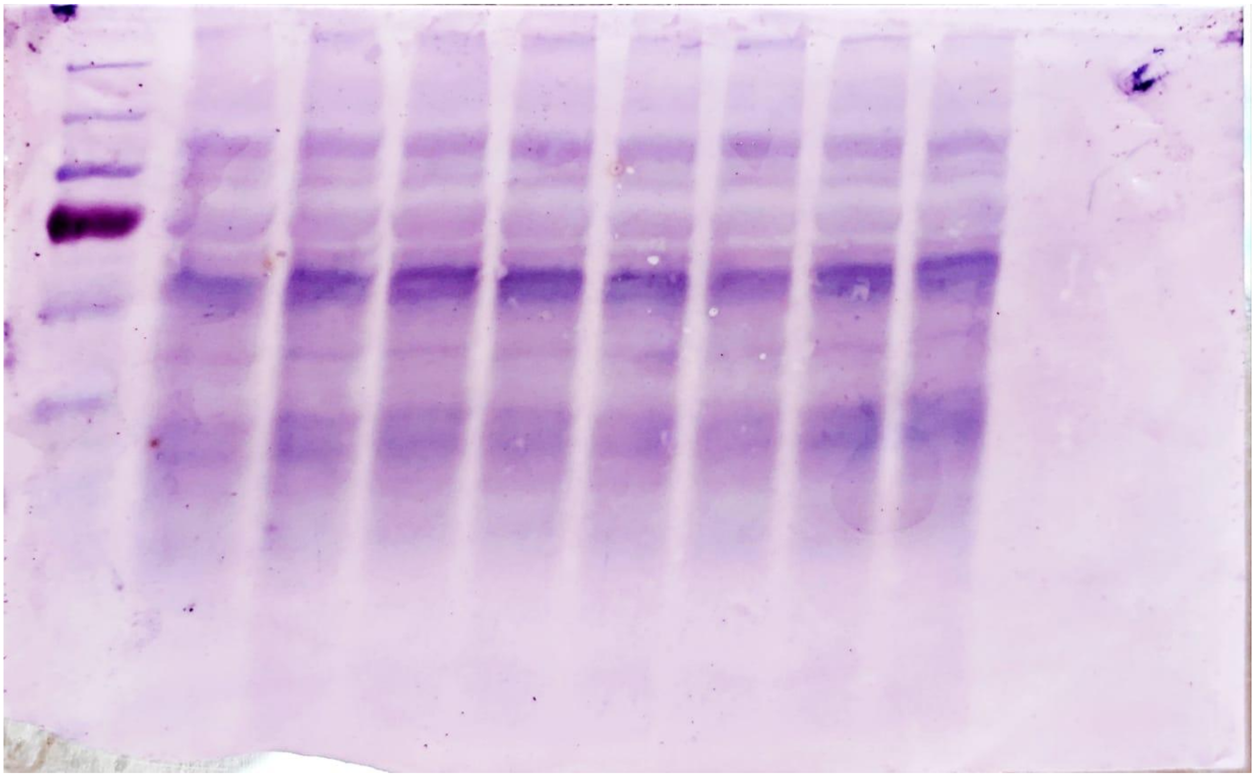

Total ERK1/2 first membrane

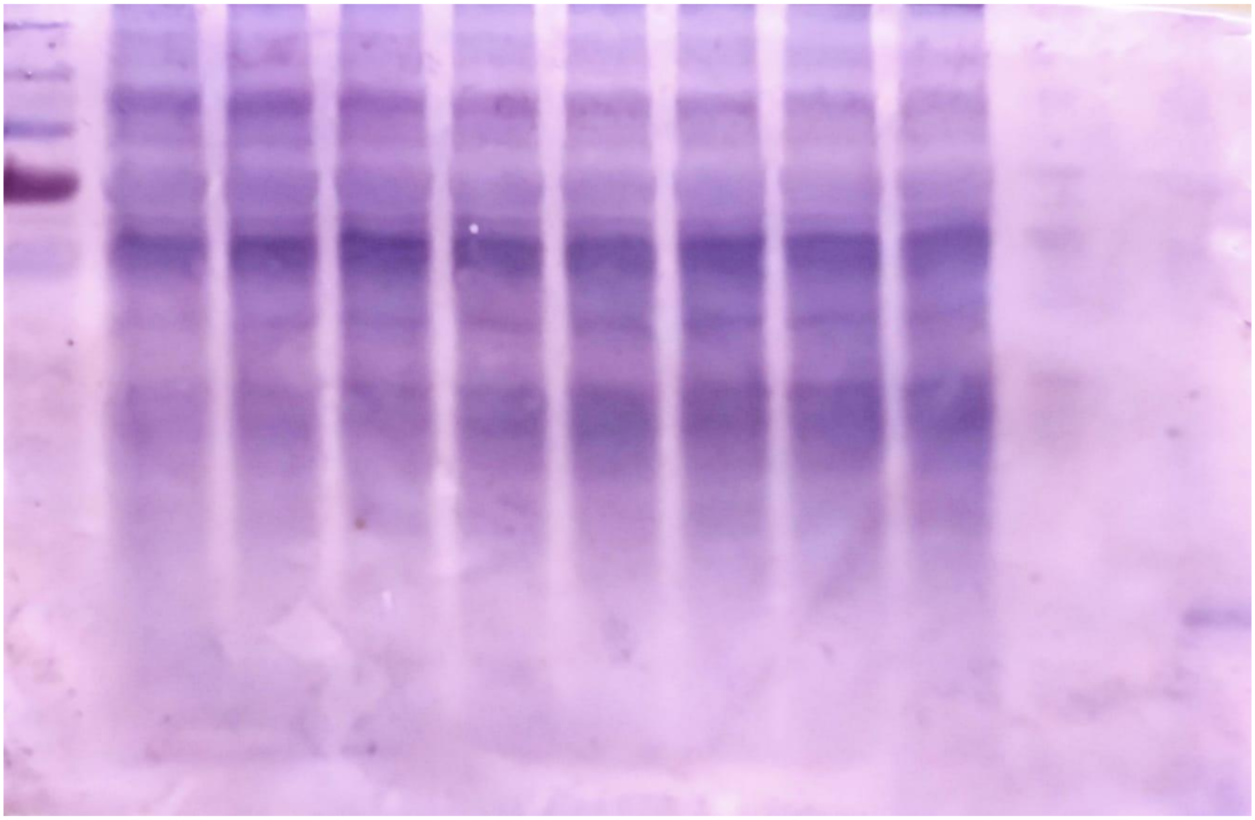

Total ERK1/2 Second membrane

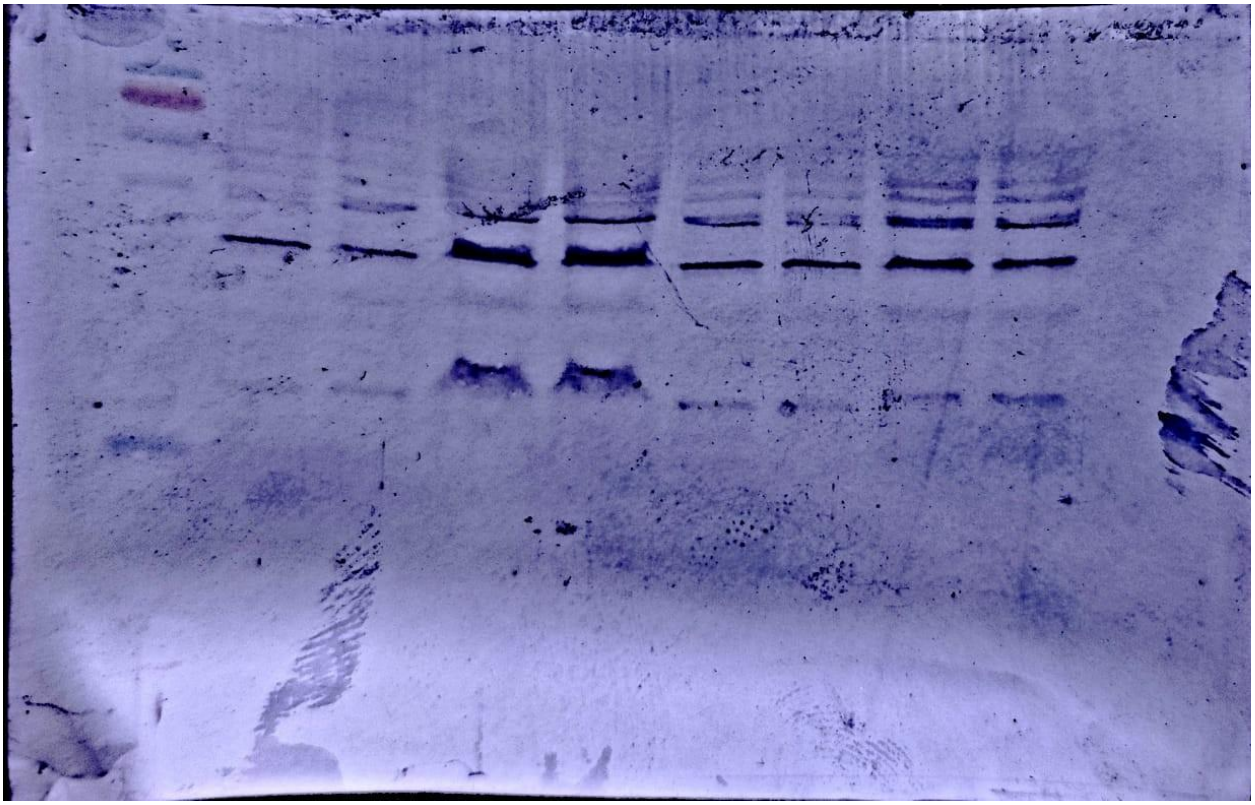

p-ERK1/2 first membrane

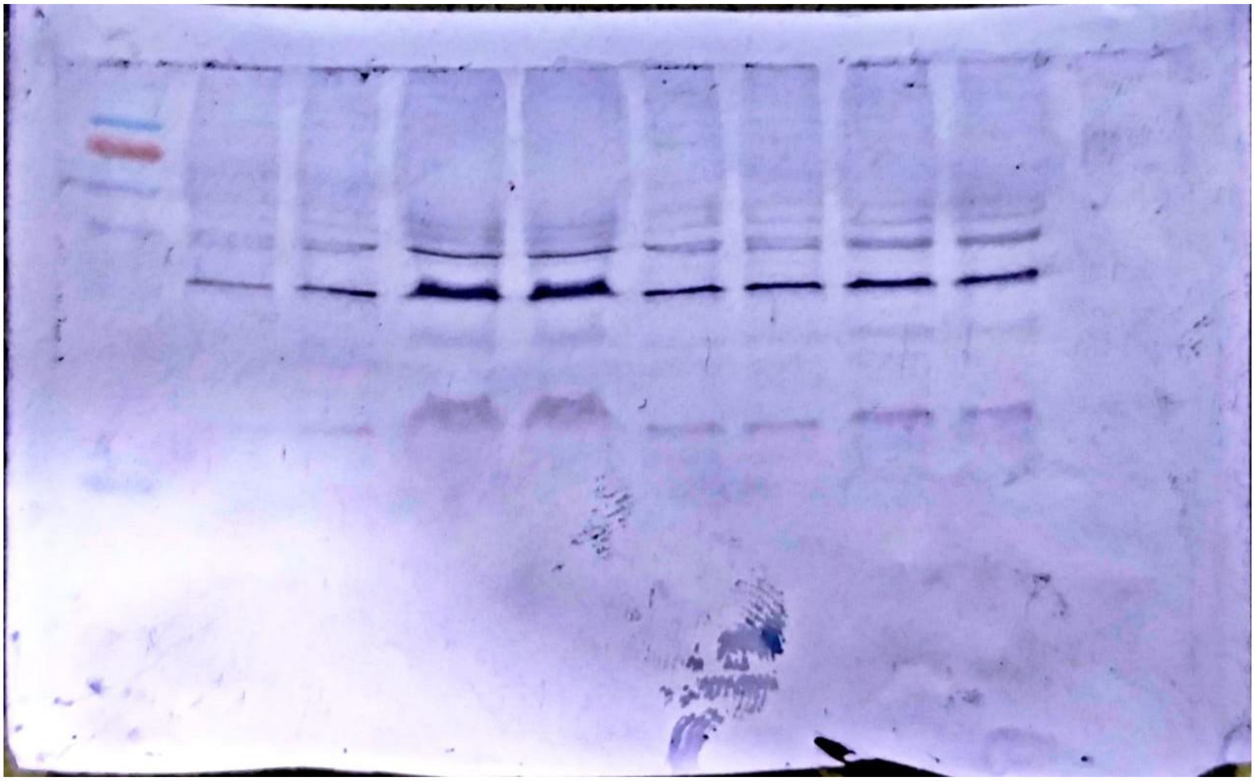

p-ERK1/2 second membrane

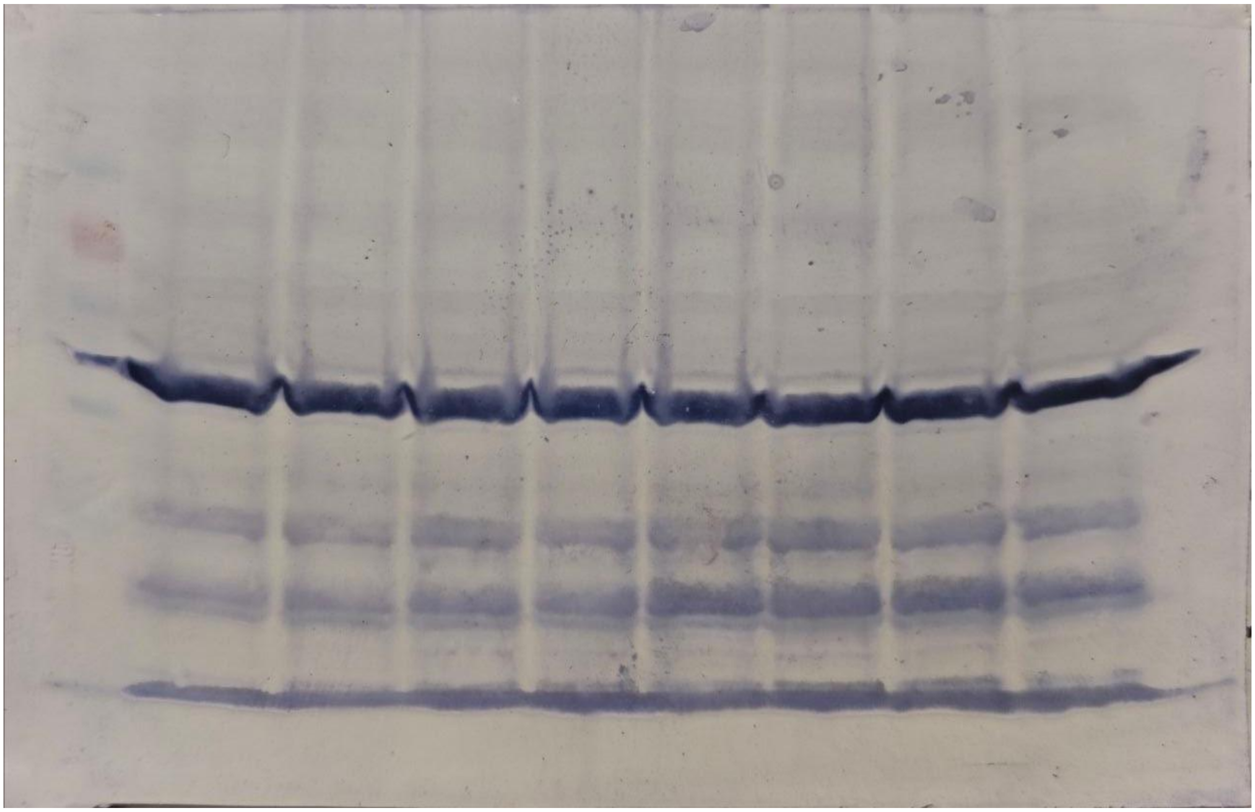

β actin first membrane

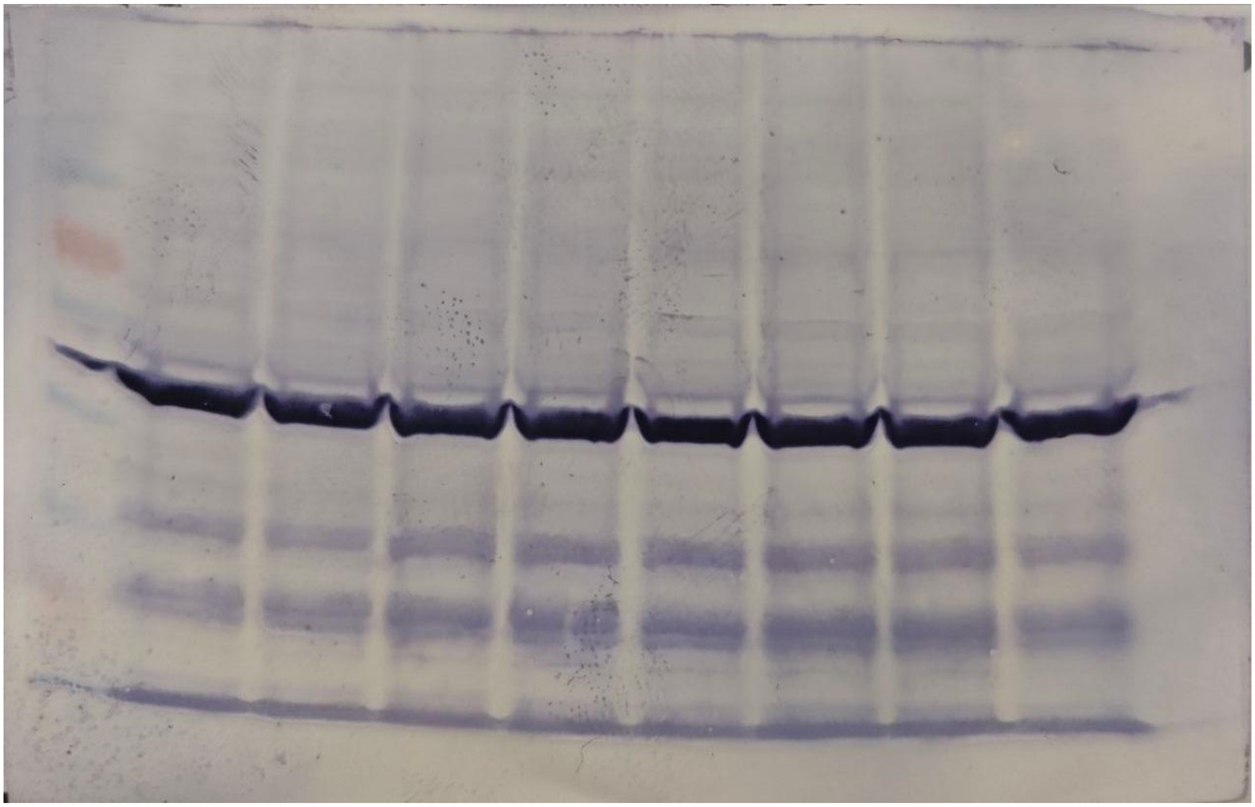

β actin second membrane
